# Engineering Microneedle Patches for Improved Penetration: Analysis, Skin Models and Factors Affecting Needle Insertion

**DOI:** 10.1007/s40820-021-00611-9

**Published:** 2021-03-16

**Authors:** Pooyan Makvandi, Melissa Kirkby, Aaron R. J. Hutton, Majid Shabani, Cynthia K. Y. Yiu, Zahra Baghbantaraghdari, Rezvan Jamaledin, Marco Carlotti, Barbara Mazzolai, Virgilio Mattoli, Ryan F. Donnelly

**Affiliations:** 1 Istituto Italiano Di Tecnologia, Centre for Materials Interface, Viale Rinaldo Piaggio 34, 56025 Pontedera, Pisa Italy; 2 School of Pharmacy, Queen’s University Belfast, 97 Lisburn Road, Belfast, BT9 7BL UK; 3 The BioRobotics Institute, Scuola Superiore Sant’Anna, Viale Rinaldo Piaggio 34, 56025 Pontedera, Pisa Italy; 4 Paediatric Dentistry and Orthodontics, Faculty of Dentistry, The University of Hong Kong, Prince Philip Dental Hospital, Hong Kong SAR, China; 5 Department of Chemical, Materials and Industrial Production Engineering, University of Naples Federico II, 80125 Naples, Italy; 6 Center for Advanced Biomaterials for Health Care (iit@CRIB), Italian Institute of Technology, 80125 Naples, Italy

**Keywords:** Insertion responsive, Implantable microneedles, Skin indentation, Transdermal microneedles, Pain management

## Abstract

Transdermal microneedle (MN) patches are a promising tool used to transport a wide variety of active compounds into the skin. To serve as a substitute for common hypodermic needles, MNs must pierce the human *stratum corneum* (~ 10 to 20 µm), without rupturing or bending during penetration. This ensures that the cargo is released at the predetermined place and time. Therefore, the ability of MN patches to sufficiently pierce the skin is a crucial requirement. In the current review, the pain signal and its management during application of MNs and typical hypodermic needles are presented and compared. This is followed by a discussion on mechanical analysis and skin models used for insertion tests before application to clinical practice. Factors that affect insertion (e.g., geometry, material composition and cross-linking of MNs), along with recent advancements in developed strategies (e.g., insertion responsive patches and 3D printed biomimetic MNs using two-photon lithography) to improve the skin penetration are highlighted to provide a backdrop for future research.

## Highlights

- Factors affecting microneedle insertion into skin are reviewed.
- The use of artificial and computational skin models for the simulation of needle insertion is summarized.
- Skin structures and models, as well as mechanical analyses, used to determine transdermal microneedle ability to insert into skin are highlighted in the review.

## Graphical abstract

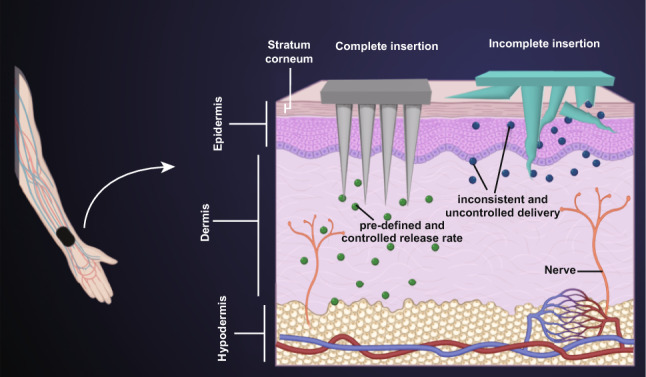

## Introduction

Drug delivery using microneedles (MNs) through the skin is an attractive route of administration [1–3]. The major advantages of MN-mediated drug delivery are the ability to deliver drugs through a large surface area, administration feasibility [4–7], avoidance of first-pass metabolism and gastrointestinal degradation [8, 9]. Drug delivery via the skin is useful either for releasing the drug into the layers of skin at the site of administration (e.g., skin abnormalities treatment and vaccination) known as dermal delivery [10, 11], or by delivering the cargo systemically through the administration from the skin known as transdermal delivery (e.g., insulin therapy) [12, 13].

Apart from the many benefits provided by MNs, a number of challenges must be overcome to achieve a clinically acceptable drug delivery device. For instance, fracture or breakage of needles during or after patch administration may change the drug release profile, which may lead to premature drug release. This problem with needle fracture may be due to inadequate mechanical strength. Debris that remains in the skin may cause harm to the surrounding tissue [14, 15]. MNs can also bend or twist while inserting in the skin [16]. Consequently, the cargo can be liberated prematurely, which prohibits controlled drug release [17].

Furthermore, MNs often suffer from insufficient skin insertion. This occurs when MNs are not able to sufficiently puncture and penetrate into the skin, which may waste the drug formulated in the MN [14, 17, 18]. At their most fundamental level, MNs must show sufficient insertion into skin and therefore must demonstrate sufficient strength to penetrate without breaking or bending during application. Although such a task appears relatively simple upon first appearance, numerous factors are accountable for adequate MN insertion, such as geometry, needle height, thickness and tip radius, base diameter, needle density, and MN material [19]. This is in addition to factors such as skin thickness and elasticity, which are unrelated to the MNs themselves but will also have an effect on the efficiency of MN insertion [20, 21]. For the clinical acceptance of MNs, it is imperative that they can reliably insert into the skin without bending, buckling or fracturing. A wide range of MN designs exists within the literature in an attempt to optimize the insertion for clinical applications [22, 23].

The present review deals with the skin penetration of MN and the employed strategies to circumvent this hurdle. Herein, an introduction on pain signal and its management is discussed, as well as a comparison between pain caused by MNs and a traditional hypodermic needle. Thereafter, skin structure, its mechanical behavior, and skin resealing are discussed. In addition, mechanical tests, skin models, and penetration tests used to assess MN insertion before clinical practice are presented. Ultimately, emphasis is given to factors affecting insertion (such as geometry, material composition, and cross-linking of MNs), which is accompanied by modern progressions in developed strategies, pedestal-based platform, to name a few, to improve MNs’ skin insertion and to pave the way for future research.

## Pain Management

Broadly stated, there are four phases in the nociception of pain: (1) transduction, (2) transmission, (3) perception, and (4) modulation [24]. Pain detection is mediated by nociceptors which are present in the skin, deep tissues, and internal organs, with the skin a densely populated area containing different types of sensory afferents [25].

Transduction is the first process of nociception, which involves the conversion of a noxious stimulus into electrical signals in the peripheral terminals of nociceptor sensory fibers [24]. Nociceptors are essentially sensory receptors located at the free endings of first-order afferent neurons (Aδ and C fibers) in the pain pathway. The larger, fast-conducting lightly myelinated Aδ fibers are activated by mechanical and thermal stimuli, which are responsible for the initial sharp pain perceived at the time of injury. Conversely, the smaller, slow-conducting unmyelinated C fibers respond to chemical, mechanical, and thermal stimuli with a high activation threshold. Such fibers therefore are associated with a longer-lasting, diffusing pain [26]. These nociceptors detect noxious stimuli and turn the stimuli into electric signals, which are then sent to the central nervous system. Both the Aδ and C fibers have their cell bodies within the dorsal root ganglion, which allow rapid transmission of the stimulus from the periphery to the spinal cord.

Transduction is followed by transmission, which includes the conduction of action potentials from the peripheral terminal along axons to the central terminal of nociceptors in the central nervous system [24]. First-order neurons carry the noxious information from the point of stimuli in the skin to the spinal cord via the dorsal root, where they synapse with the second-order neurons in the dorsal horn. The dorsal horn is divided into laminae (called Rexed laminae), whilst the C fibers terminate in lamina II and the Aδ fibers terminate in laminae I and V [27]. These second-order neurons then cross over to the other side of the spinal cord before ascending to the brain. There are two major pathways that carry nociceptive signals from the spinal cord to the brain, the spinothalamic and spinoreticular tracts, which are located in the anterolateral white matter of the spinal cord [24]. In the first pathway, second-order neurons ascend from the contralateral spinothalamic tract before terminating in the ventral posterolateral nuclei and central nuclei of the thalamus, where they synapse with third-order neurons, which play a major role for processing somatosensory information [27].

**Fig. 1:**
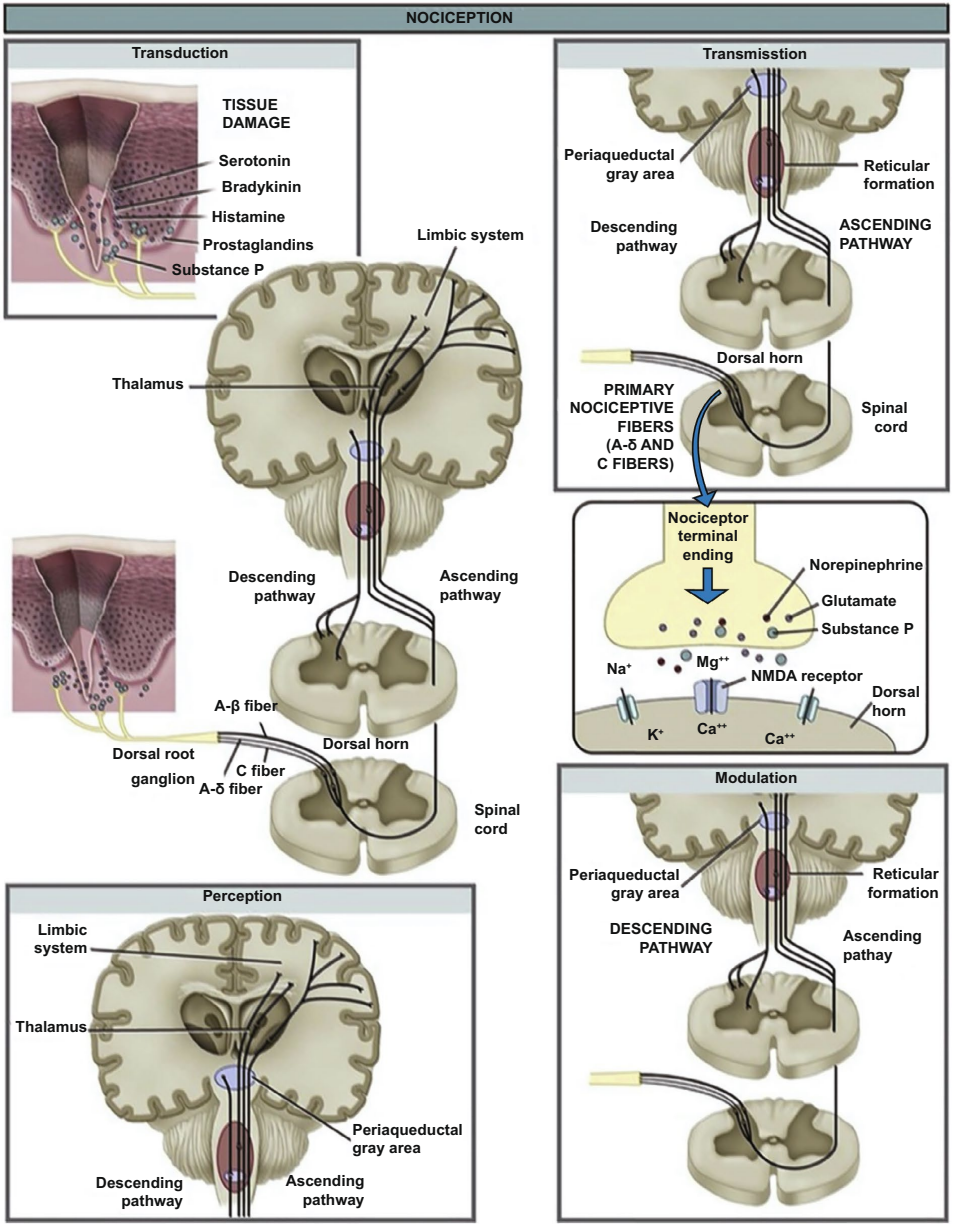
The basic route of pain transmission upon noxious stimuli. There are the four phases (transduction, transmission, perception, and modulation) in the nociception of pain. Reprinted from Ref. [30] with permission from Springer

The third-order neuron then projects via the posterior limb of the internal capsule to terminate in the ipsilateral post-central gyrus (primary somatosensory cortex); this pathway is involved in the localization and intensity of the painful stimulus [28]. Simultaneously in the spinoreticular tract, fibers ascend the contralateral cord to the reticular formation of the brainstem before running up to the thalamus, hypothalamus and ultimately the cortex; this pathway is responsible for the emotional aspect of pain [27]. Hence, pain signals from the skin will terminate in the cortex to interpret the pain sensation. The perception of pain constitutes the third process of nociception. Modulation is the final process in nociception, which is an adaptive process involving both excitatory and inhibitory mechanisms, thereby altering the perception of pain [24]. Nociceptive signaling may be augmented by central mechanisms of hyperalgesia (exaggerated pain following noxious stimuli) or allodynia (pain from a typically harmless stimulus), and in contrary, nociceptive signaling may also be decreased by endogenous analgesia systems [29]. The basic diagram of pain transmission is presented in Fig. 1.

There are several clinical studies reporting that MNs elicit less pain when compared to the use of hypodermic needles [31–36]. Kaushik et al. evaluated the pain sensation of 12 healthy volunteers between 18 and 40 years of age following the use of a silicon MN array containing 400 needles with a length of 150 µm. The subjects reported substantially less pain with MN application than a 26-gauge hypodermic needle. Likewise, Bal et al. examined the pain generated by stainless-steel MNs with length less than 550 µm in 15 volunteers, aged between 21 and 30 years, and found very low pain scores in all subjects, with no significant differences among MNs of different lengths or shapes [30, 35].

Conversely, in a randomized, single-blinded human clinical trial conducted in 15 healthy adult subjects, a local anesthetic (lidocaine) was administered using hollow borosilicate-glass MNs and 26-gauge hypodermic needles. A reduced pain sensation in both the forearm and dorsum of the hand was reported with MNs, with a similarly rapid onset and efficacy as 26-gauge hypodermic needles [32]. In a single-blind study involving 12 subjects comparing pain and sensation following application of a 25-G hypodermic needle and two MN arrays (36 needles of 180 and 280 µm in length), pain intensity and sensory perception were evaluated using a visual analog scale (VAS) and an adapted McGill Pain Questionnaire Short Form, respectively. The VAS pain scores showed that the participants experienced significantly less pain with 180 and 280 µm MNs than the hypodermic needle. Furthermore, results from the questionnaire showed that the participants perceived greater “sharp” and “stabbing” sensations with the application of hypodermic needle, while “pressing” and “heavy” sensations were experienced with the MNs [34].

In another clinical study involving 10 healthy subjects (18–40 years of age), Gill et al. similarly reported that stainless-steel MNs were significantly less painful than a 26-gauge hypodermic needle. Among the factors investigated (MN length, number of MNs, MN tip angle, thickness and width), significant pain reduction was observed with a decrease in MN length and number of MNs. A threefold increase in the MN length from 480 to 1450 µm resulted in a sevenfold increase in pain perception from 5 to 37%, while a tenfold increase in the number of MNs only resulted in a relatively small 2.5-fold increase in pain. Hence, optimizing MN length is the crucial factor to minimize pain sensation with application [31].

In a clinical study, the use of hollow borosilicate-glass MNs for the injection of sterile saline in 15 human subjects was evaluated. It was concluded that the infusion of a few hundred microliters of fluid is commonly performed in clinical practice, and up to 1 mL of saline delivery using a MN array was less painful than injecting the same volume via a hypodermic needle [33]. Lower pain perception was observed with a short MN array, lower flow rate and infusion using hyaluronidase compared to sterile saline. In another level 3 clinical study, levels of pain (and bruising) experienced by 20 human subjects, aged between 26 and 60 years, injected with a 30-gauge hypodermal needle versus 33-gauge silicon MNs were compared in procedures that involved multiple injections. MNs were considerably less painful with a lower risk of bruising for non-surgical facial cosmetic procedures that involve multiple injections to the face [36].

## Skin Structure and Its Mechanical Behavior

**Fig. 2:**
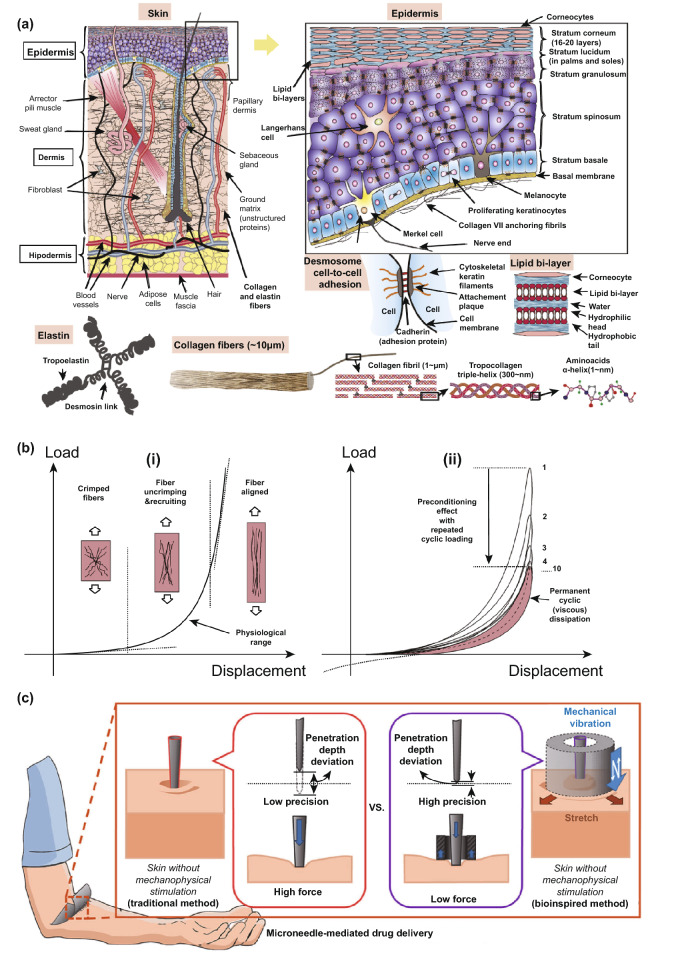
**a** The structures of the skin and its layer. **b** Stress–strain behavior of soft tissues during tensile tests: (i) preconditioned hyperelastic behavior and (ii) preconditioning and viscous effects. Reprinted from Ref. [39] with permission from Elsevier. **c** Impact of changing skin resistance to microneedle penetration. Reprinted from Ref. [40] with permission from Elsevier

The skin is a multilayered organ that acts as a critical barrier, protecting the organism from chemical, physical and biological threats coming from the environment. In addition, it maintains homeostasis and is involved in sensory mechanisms and metabolic processes. The skin is divided into three layers (epidermis, dermis and hypodermis), and each layer is composed of different cells and proteins of the extracellular matrix (ECM), as well as by specific structures characterizing each layer, including nerves, blood vessels, hairs and glands (Fig. 2a) [37]. Collagen is the principal protein of ECM and accounts for 75% of the dry weight of skin [38]. Moreover, the skin composition depends on the location and varies with gender, race, age and illnesses [39].

Constituents of the ECM are structural (i.e., collagen, elastin and fibrillins) and specialized proteins (i.e., fibronectin, integrins and several laminins) [37]. In addition, 80% of the volume of the skin and 20% of its dry weight are composed of ground matrix or substance including proteoglycans [41]. Proteoglycans are a ubiquitous family of macromolecules containing a core protein and glycosaminoglycan side chains bound to the core, similarly to hyaluronic acid [42].

The epidermis is mainly composed of keratinocytes, which produce keratin (a protein constituting the filaments of the cytoskeleton, which has protective functions), and 5% of other cells such as Langerhans cells, Merkel cells and melanocytes [37]. The epidermis is made up of 5 sublayers according to the degree of keratinization of the cells: *stratum corneum, stratum lucidum, stratum granulosum, stratum spinosum and stratum basale* [43]. At the basal layer of the epidermis, the keratinocytes are proliferative, and as cells mature, they migrate toward the surface and lose their proliferative potential. The cells undergo programmed death, fuse together, lose their nuclei, release their glycolipids into the intracellular space and become corneocytes in the *stratum corneum*, dominated by keratinized cells bonded by desmosomes and stacked as layers (Fig. 2a) [44].

The intermediate layer of the skin, the most important thermal and mechanical unit of this tissue, is the dermis, which has a thickness between 0.6 and 3 mm (thinner on the eyelids and thicker on the back, soles and palms). The dermis is linked to the epidermis through the basement membrane by the dermo-epidermal junction, which is made of hemidesmosome structures that have a thickness of between 65 and 175 nm. Histologically it is simple to identify two layers in the dermis, due to the different cellular density, fiber distribution, vascular and nerve networks. The lowest and thicker layer is the reticular dermis that has a quasi-planar and dense distribution of collagen fibers, which become less dense in the deeper areas. The uppermost layer is the papillary dermis, which has an undulating system in which collagen fibers are less numerous and structured in space [37]. The fibers of the principal type I collagen associate with collagen III and run mainly in a parallel plane to the skin surface, with some excursion out of this plane and some cross-linking. Because of this structure, mechanical properties change when measured from different preferential directions, and thus, the dermis is usually modeled as an orthotropic material [39]. The most abundant cells in the human dermis are the fibroblasts. These cells continuously create both collagen and elastin fibers in the ECM in response to mechanical signals (forces or deformations) that cells receive from the environment (i.e., mechanosensing) [45, 46].

The layer under the dermis is the hypodermis, which includes blood vessels connected to the dermis and cells such as fibroblasts and macrophages, but mainly adipocytes, which have the function of storing energy and providing thermal insulation. From a mechanical point of view, the hypodermis behaves as a damper of shock impulses [47]. It is connected to the dermis by collagen and elastin fibers and to the deep fascia by fibrous bands in a floating manner. This configuration allows mechanical isolation from other body components [39].

As previously described, collagen and elastin are important constituents of the skin tissue, and they are the main contributors to its mechanical response. Collagen in the dermis accounts for 25% of the volume and 70% of the weight [39]. The spatial scale is well understood for this protein (Fig. 2a): aminoacids including glycine, proline and hydroxyproline have a size of around 1 nm and form α-helixes which enlace in the tropocollagen. Then, microfibrils (~ 1 µm in diameter) are formed from multiple tropocollagen molecules covalently cross-linked in parallel by aldehyde bonds. Finally, microfibrils associate in fibers which have a “wavy” pattern (~ 100 µm in diameter) [39].

Elastin takes about 1% of the volume, and up to the 2–4% of the dry weight of the dermis and morphologically elastin may be compared to a 100-nm-thin strand attached to the fibers of collagen, which forms an intricate 3D network [48]. The basic component of this protein is the elastin fibril, coiled and highly stretchable, made from tropoelastin molecules. Tropoelastin is cross-linked in quadruplets by desmosine links (Fig. 2a) [49]. Collagen and elastin are embedded in an amorphous highly hydrated ground substance, which is a thixotropic semi-fluid with high viscosity [50].

The overall mechanical response of the skin depends on the behavior of each of its constituents when exposed to a wide range of strains. When uniaxial stretching is applied to the skin, the stress–strain relation assumes the behavior showed in Fig. 2b(i). As can be seen, at low strains tissue behavior is prevalently isotropic with main response contributions provided by elastin and ground matrix, whereas collagen fibers remain undulated and crimped. The second zone in the curve is nonlinear due to fibers of collagen that progressively begin to unfold and align with increasing load. The resulting average stiffness increases as more local fibers un-crimp. At large strains, collagen fiber stretching along the load direction dominates, the skin is stiffer and the curve has almost a linear behavior (Fig. 2b(i)) [51].

From these studies, it is evident that elastin, collagen and ECM are largely involved in the response to stress and strain. Elastin is a protein essential for the elasticity and resilience of the skin. Its Young’s modulus is ~ 100 kPa [49]. The mechanical role of elastin is manifested in diseases such as *cutis laxa*, in which skin is characterized by abnormal wrinkling and laxity due to structural defects in the extracellular matrix or in the synthesis of elastin [52].

Collagen is responsible for the stiffness of the skin; indeed, its elastic modulus is about 1.0–10 GPa [53], many orders of magnitude higher than that of elastin. While in tissues, ligaments and tendons, collagen fibers form in a parallel alignment respect to each other, the behavior of these tissues can be modeled as transverse-isotropic materials. In skin and blood vessels, they form a 3D network of coiled fibers organized in one or two preferred directions, causing the anisotropic behavior of the skin [51, 54]; thus, orthotropic models can be used. The mechanical role of collagen is manifested in diseases such as the Ehlers–Danlos syndrome characterized by skin hyperelasticity among other defects due to mutations in encoding pro-collagen chains [55]. Finally, the ground substance starts to provide a mechanical contribution when the tissue is compressed or is under shear stress [39, 56].

Collectively, skin tissue is considered to be an anisotropic [51, 54] and viscoelastic material [56]. However, its biomechanical properties highly depend on skin thickness, age, illnesses, race, and environmental factors. For example, when comparing skin on the forehead with skin on the ventral forearm, forehead skin is thicker, stiffer, less tense and less elastic [57]. Elastin fibers begin to degrade between 30 and 70 years of age, with a decrease in the capability of potentially recovering deformations [58]. Collagen half-life instead varies with the specific tissue in question; in the skin, the duration was measured being 15 years [59]. Furthermore, diseases such as cutis laxa acrokeratoelastoidosis are characterized by a loss of elastic tissue, which will in turn reduce the elasticity of skin, affecting its biomechanical properties [60]. Berardesca et al. [61] investigated the role of race on skin biomechnics and found that racial differences in skin physiology exist and are mainly related to the role of melanin present in races with darker skin. In terms of elastic recovery, Hispanic skin showed the greatest recovery on dorsal and volar sites when compared to white skin (*p* < 0.05). Viscoelasticity was also found to be significantly different between white and Hispanic skin across both dorsal and volar sites [61]. Alterations in environmental humidity as well as temperature can have a significant influence on the mechanical properties of the *stratum corneum*. Typically, its Young's modulus decreases with increasing skin hydration because water induces a plasticization effect on its structure [62].

As mentioned above, skin is a viscoelastic tissue, which highly depends on the strain rate, entity and temperature. The main contributor to viscoelasticity is the interaction of the matrix with the collagen fibers [39, 56]. As can be seen from Fig. 2b(ii), when loading and unloading are repeated consecutively), the skins response is characterized by a preconditioning effect due to a temporarily irreversible adaption to cyclic load of the proteins and fiber arrangement. Moreover, a hysteresis effect or viscous dissipation can be distinguished in the curve, caused by the presence of unstructured proteins in the ECM and by a partial anisotropic effect due to the fibers (Fig. 2b(ii)) [63].

As the fibers entrapped in the ECM are the principal mechanical component of the skin, the tissue can be modeled as a fiber-reinforced material, with more or less complicated models; frequently, an exponential stress–strain curve is associated with the reinforcement part of the tissue, to take into account the progressive alignment of the collagen fibers as well as elastin role, whereas the ground matrix is often modeled with a more classical behavior, as the Neohookean [39].

Understanding the mechanisms underlying skin composition and mechanics is important to improve MN penetration in humans. While MN insertion has been focused on the MNs themselves, altering parameters such as material, MN shape, tip and base diameters, height, density, indentation forces, minimum curvature radius to pierce the *stratum corneum*, etc. [64, 65, 66, 40], the behavior of the skin following MN application has not been fully explored. The physically robust *stratum corneum* is the layer responsible of the stiffness during the application of a MN array. The dermis, with its collagen and elastin fibers in the matrix, provides the skin strength and flexibility during skin puncture, with, as previously shown, an increase in skin stiffness as fibers straighten. The hypodermis significantly contributes to tissue deformation, but its contribution is typically neglected [39]. However, further knowledge is necessary in regard to the mechanical behavior of skin during penetration in order to design MNs in a rational way before their fabrication, particularly to determine the most effective MN geometries, application methods and to produce a uniform and reproducible MN penetration.

In this regard, Kim et al. explored for the first time the effects of changing skin resistance to MN indentation by inducing the application of external stimuli to the tissue. They developed a mosquito-inspired skin piercing mechanism to insert a MN with high precision and low force (Fig. 2c). In particular, they applied to the skin a uniaxial/equibiaxial stretching of 0–20% (static stimulus), and through a piezoelectric actuator, they transmitted vibration with an amplitude of 1–10 µm and a frequency of 1–1000 Hz (dynamic stimulus) during MN insertion. They found that the static stimulus they applied mainly affected the precision of MN insertion, while the dynamic stimulus controlled skin resistance to MN penetration [40]. These findings may lead to a greater understanding of skin biomechanics during piercing.

## Skin Resealing

It is important to assess the potential impact of disrupting the *stratum corneum* following MN application and subsequent removal of the MN. Although it is necessary for MN-induced pores to remain open during the application period, it is also imperative for these pores to reseal in a timely manner [13]. In doing so, this minimizes the risk of microbe infiltration, which could lead to potential infections. Using noninvasive biophysical tools, such as transepidermal water loss (TEWL), several research groups have examined the skin resealing time following the application of a wide range of MN designs and geometries. Using MNs manufactured from stainless steel, one such study examined the effects of increasing MN length (500–1500 µm) and needle number (10–50 needles) on skin resealing time using both occlusive coverings and non-occlusive conditions in healthy human adult subjects [67]. Non-occlusive data showed that changing the MN geometry had no effect on skin resealing time. In this instance, a 26G hypodermic needle was used as a positive control. Interestingly, skin resealing time for both the positive control and the different MN geometries was observed to be within 2 h of application. Under occlusive conditions, it was found that doubling the needle length resulted in a sixfold increase in skin resealing time. In addition, a fivefold increase in the number of needles corresponded to a tenfold increase in barrier resealing time. The authors suggested that a plausible reason for the increase in resealing time is the reduced TEWL under occlusive conditions. This is based on previous studies which have shown that the resealing of the *stratum corneum* is controlled by the formation of a water gradient when the skin has been breached [68]. Therefore, occluding the skin eliminates this gradient, thus over-hydrating the *stratum corneum* and slowing the healing process [69]. Importantly, following the removal of the occlusive barrier, the pores closed rapidly. As a result, the fast resealing time following the removal of a MN or an occlusive patch minimizes the chance of infection at the application site using a “poke-and-patch” approach.

The resealing time of solid, wet-etched silicon MNs has also been tested. In this study, twelve human subjects received single-blinded insertions of two MN arrays (180 and 280 µm in height) and a 25G hypodermic needle. TEWL was used to measure the level of disruption to the *stratum corneum* following application of these devices. This was measured immediately after application and over-specified time points over a period of 24 h. As expected, TEWL increased significantly post-application for both MNs and hypodermic needle. Perhaps more importantly, TEWL returned to baseline after 24 h for all three devices. This observation confirmed that despite the creation of numerous pores following the application of silicon MNs, the skin barrier can still reseal in a timely manner [34].

**Fig. 3:**
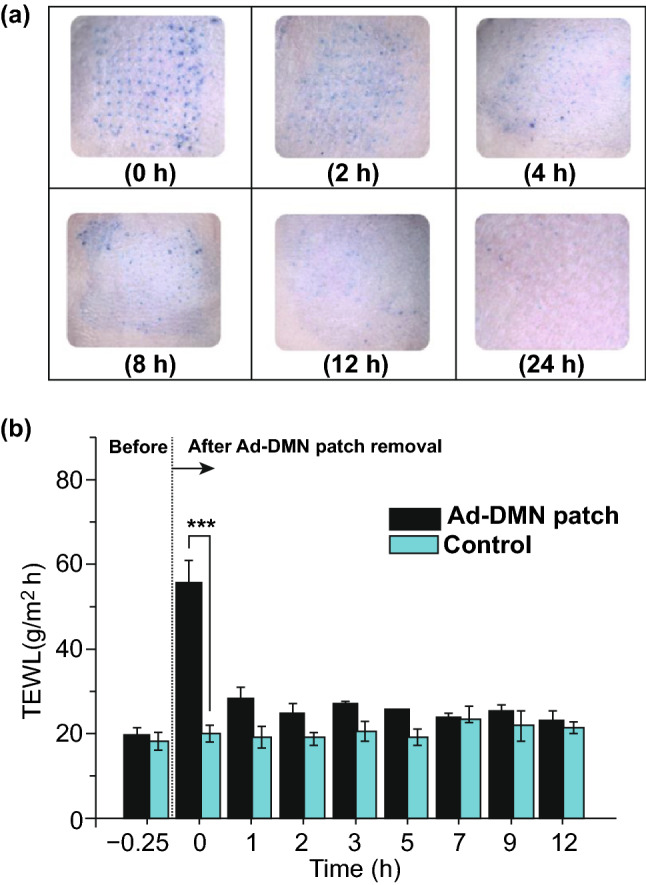
**a** Light microscope images of mice skin over a 24-h time frame following the removal of an alpha-arbutin-loaded dissolving MN after a 2.5-h application period [71]. **b** Transepidermal water loss (TEWL) values to examine the skin resealing kinetics before and after the application of Ad-DMN over 12 h. Reprinted from Ref [72] with permission from Willey

Minimal skin barrier disruption has also been demonstrated using hollow MNs. In one particular study, gold-coated Radel® R hollow MN arrays, composed of 36 needles, 1.2 mm in length, were used. These MNs were applied to murine skin in vivo, with TEWL measured over a 24-h period. TEWL values increased significantly after MN application to the skin, thus confirming that the skin barrier had been disrupted. As observed in previous studies using solid MNs, TEWL values returned to baseline after 24 h, proving that the disruption to the *stratum corneum* is minimal and reversible [70]. More recently, the resealing time of dissolving hydroxypropyl methylcellulose/polyvinylpyrrolidone (HPLC/PVP) MNs loaded with alpha-arbutin was investigated in vivo [71]. In this study, 11 × 11 MNs, with height (520 ± 1.7 µm) and base width (294 ± 3.85 µm), were applied to living mice for 2.5 h after which the application site was imaged under a microscope for 24 h as shown in Fig. 3a. Importantly, after 24 h, the number of visible microchannels decreased by ~ 94%, thus showing that in vivo, skin was able to naturally repair. In addition, no signs of infection were observed over the duration of the experiment, indicating that this MN device could be safely applied and removed from the skin [71]. In another study using dissolving MNs for the treatment of wrinkles, the skin resealing kinetics after insertion of a dissolving MN containing adenosine (Ad-DMN) was assessed using TEWL [72]. In this instance, the Ad-DMN fully dissolved in the skin situated below the right eye of 27 healthy women volunteers after 1 h of application. This site resulted in a significant increase in TEWL compared to the left eye with no treatment (control). As shown in Fig. 3b, this value decreased back to a similar level to that of intact skin within 7–12 h, further proving that resealing of the *stratum corneum* occurs within a reasonable timeframe [72].

Using the more recently developed hydrogel-forming MN design, the effects of skin barrier integrity and resealing time after multiple MN applications were examined. In this particular study, 11 × 11 hydrogel-forming MNs composed of Gantrez® S-97 and PEG 10,000 with the following dimensions: 600 µm needle height, 300 µm base width and 300 µm interspacing, were applied to 11 healthy human volunteers each day for five days, with TEWL measurements taken from the MN application site before and after application each day [73]. As expected, immediately after the MNs were removed, TEWL increased between 3 and 10 times the baseline values. Perhaps more importantly, TEWL returned to normal when measured 18 h post-MN removal. However, based on previous studies using the same MN design, the authors suggested that the time required for the *stratum corneum* to reseal is actually much shorter than 18 h [74]. Therefore, this study proved that repeat application of MNs over a fixed period does not disrupt, or indeed, cause long-term damage to the skin’s barrier function.

Although the kinetics of MN pore closure is variable and dependent on animal type, the plethora of studies involving human subjects indicates that MN application does not adversely affect the long-term barrier properties of the skin. In addition, there is a growing body of evidence, proving that MN application does not result in systemic reactions, owed in part to the skin resealing time. Therefore, these studies help bring MN technology one step closer to commercialization.

## Mechanical Analysis

The mechanical properties of MNs (elastic modulus and facture force) must be analyzed to ensure that they will not bend or fracture during skin insertion tests. Mechanical testing is used to measure the maximum axial force which causes MNs failure. By enhancing both Young’s modulus of the material and base diameter, the amount of yield force is increased. Additionally, the failure force enhances with decreasing the needle length, since the critical buckling (lateral deflection) load of a column decreases with the increasing column length [75, 76]. With some generalizations, these explanations are proved analytically by referring to *Euler’s formula*, the equation of critical buckling load [77]:

5.1 $$ P_{{{\text{cr}}}} = \frac{{\pi^{2} EI}}{{\left( {K L} \right)^{2} }} $$

where $$P_{{{\text{cr}}}}$$ is the critical load, $$E$$ is Young’s modulus, $$I$$ is the second moment of area of the cross section of the needle, $$L$$ is the total length of the needle, and $$K$$ is the effective length factor. $$K$$ is related to the boundary conditions of the column. Since a needle can be considered as a fixed-free column or fixed-pinned column, the corresponding effective length factor is $$K = 2$$ or $$K = 0.699 $$, respectively [78]. However, tests confirm that $$K = 0.699$$ better fits the experimental data [79]. Clearly, the critical load is directly related to Young’s modulus value and related inversely to the square of the length.

Failure force measurements acquire significance only when compared to indentation forces: the force required to insert MNs into the skin was shown to depend on the interfacial needle area at the tip. The indentation force varies linearly with the interfacial area of the needle tip [80].

To overcome skin elasticity, the applied load on the MN tip must be higher than the resistive force, approximately 0.03 mN or 3.18 × 10^6^ N m^−2^ [20, 21]. The safety factor, defined as the ratio of failure force to indentation force, has to be determined based on the aforementioned mechanical evaluations. The safety margin decreases as MN length increases. To avoid MN mechanical failure during insertion, the safety margin must be greater than unity [80, 81].

**Fig. 4:**
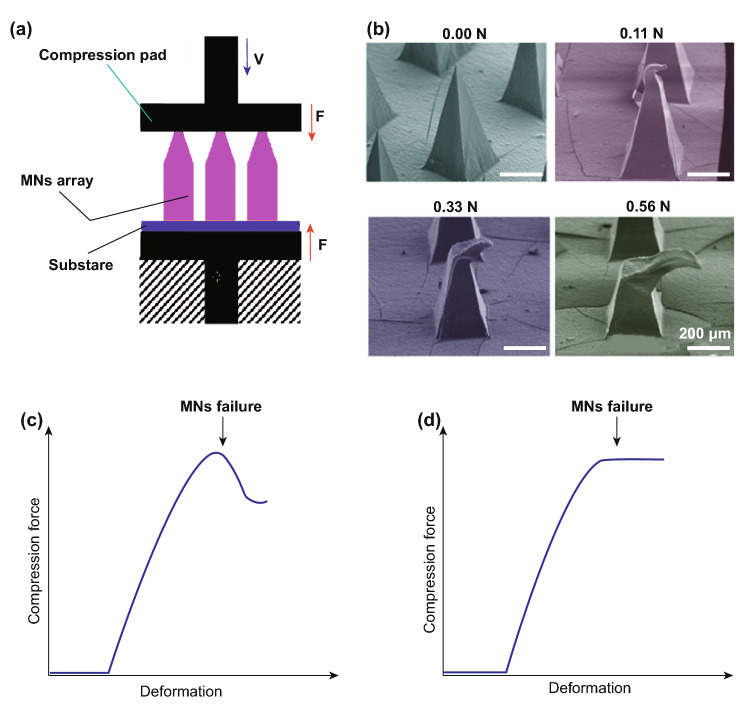
**a** Schematic illustration of the compression test for a single microneedle (MN). **b** SEM images of MNs after applying different compressive forces. Reprinted from Ref. [84] with permission from MDPI. **c** MNs failure with suddenly dropped force. **d** MNs failure with saturated force

Different mechanical tests may be explored to evaluate the mechanical properties of MNs. Among them, the compression test is mostly employed as it mimics the insertion of MNs into skin (Fig. 4a, b). In this test, an axial load testing machine, equipped with load cell and displacement gage, is implemented. A compression load normal to the substrate and parallel to the MN longitudinal axis applied to an array of MNs as shown in Fig. 4a. The compression pad’s velocity is adjustable, and the longitudinal deformation rate of the MN array is controlled. Considering the equipment’s capacities and the desired accuracy, this rate can be adjusted from 1 [82] to 500 µm s^−1^ [83]. Low deformation rate increases the extracted information resolution. By plotting the specimen’s deformation vs applied load, the compressive failure force can be determined. As shown in Fig. 4c, d, the force–displacement curve suddenly decreases when the force or force saturation point is reached [83]. In both cases, the maximum applied load is considered as the failure load. Materials with higher Young’s modules show failure load at lower displacement in compression tests.

**Fig. 5:**
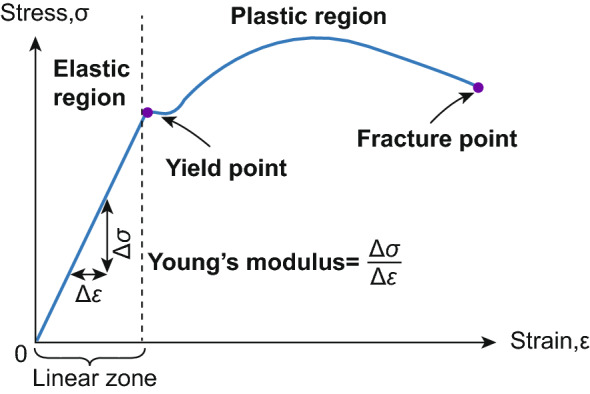
General illustration of the stress–strain graph

The stress–strain diagram of the MN can be deduced from this information in a simplified manner using the following equation [85]:

5.2 $$ \sigma = \frac{{F_{{\text{c}}} }}{A} $$

where $\sigma$ is the applied stress, $F_{{\text{c}}}$ is the compressive force, and $A$ is the sectional area of the testing substance. For a constant deformation rate, the strain is measured using the following equation:

5.3 $$ \varepsilon = v \Delta t $$

where $\varepsilon$ is the strain, $v$ is the downward speed of the testing machine’s probe, which is equal to the specimen’s deformation rate, and usually is constant, and $\Delta t$ is the elapsed time. The $\sigma - \varepsilon$ plot is a straight line in the elastic area, i.e., before the maximum applied load, and is based on Hooke's law:

5.4 $$ E = \frac{\sigma }{\varepsilon } $$

where $E$ is the well-known Young’s modulus [86] (see Fig. 5).

**Fig. 6:**
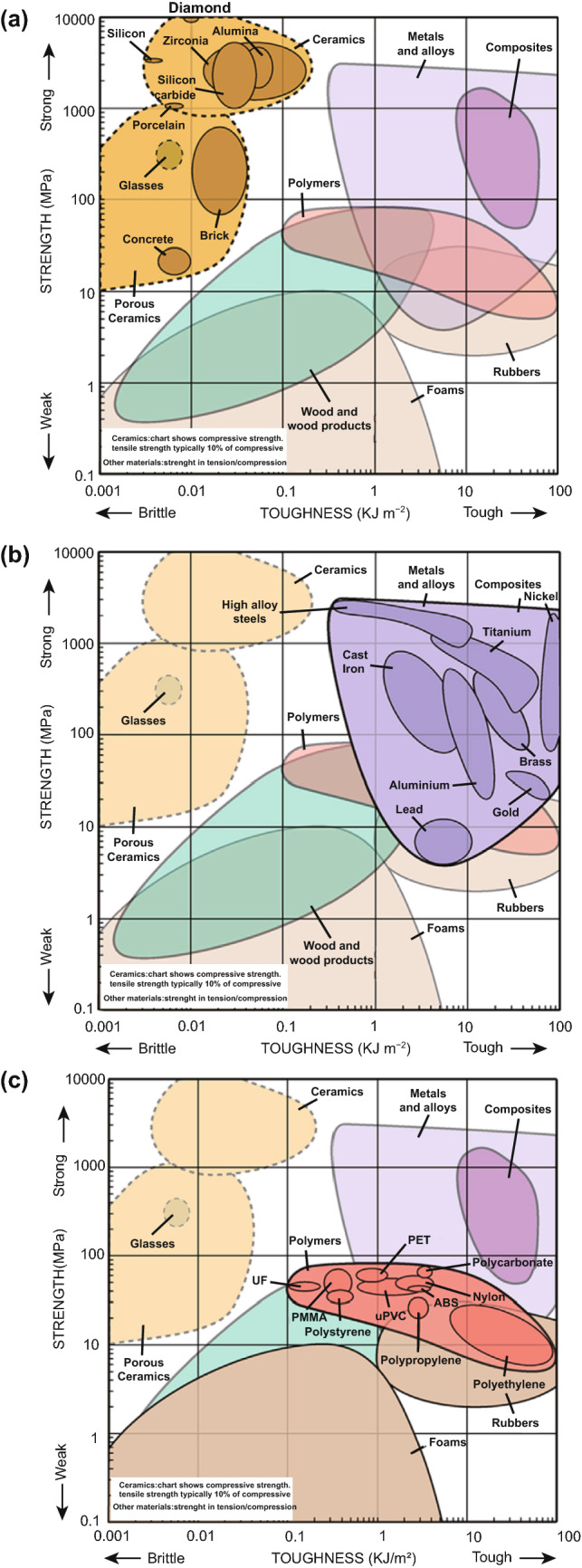
Strength versus toughness for **a** ceramics, **b** metals and **c** polymer materials, which is used to fabricate microneedles (MNs). The diagram indicates the strength (MPa) and toughness (kl m^−2^) ranges for several materials used in the MN fabrication process. For MNs to successfully insert into the skin, materials should be both strong and tough to withstand natural elasticity of skin. Weak and brittle materials are unlikely to insert without bending or breaking, potentially leaving MN material within the skin. Reprinted from Ref. [87] with permission from Elsevier

In addition to Young’s Modulus, Larrañeta et al. have addressed another considerable characteristic which affects MN insertion and has presented a comprehensive graph to compare strength and toughness for different types of materials (Fig. 6) [87]. In this graph, moving along the y axis, materials have higher strength and show more resistance to deformation and forming cracks. On the other hand, moving along the *X*-axis, materials become tougher with more fracture resistance during deformation. Therefore, needles need to be strong enough to resist deformation, and consequently, the formation of initial fracture points needs to be tough enough to prevent the increment of fracture points that may complete the fracture, resulting in breakage of the MNs. From mechanical point of view, materials located at the top-right of the graph are more convenient for fabrication of MNs. This diagram clearly illustrates that metals and their alloys produce the strongest and toughest MNs (though there is a wide range in strength/toughness values when comparing specific metals), whereas wood and foams, as the weakest and most brittle materials, are largely unsuitable for MN fabrication. These strength/toughness values combined with their established use in healthcare and good biocompatibility mean that metal MNs are an attractive material for MN fabrication [87].

## Skin Models: An Overview on In Vitro and In Vivo Models

Human skin is composed of three basic layers, epidermis (0.05–0.2 mm thick), dermis (1.5–3 mm thick) and subcutaneous tissue, providing a total thickness of around 3 mm [88]. The external layer of the skin, epidermis, is thinner in the eyelids and thicker in soles and palms. For efficient drug delivery, the length of the MNs should be designed to allow the release of drugs in the viable epidermis or very top of dermis, where drug binding, metabolism and active transport take place [89], but not exceed the thickness of the epidermis to prevent the MNs reaching the pain receptors deep in the dermis [14].

**Fig. 7:**
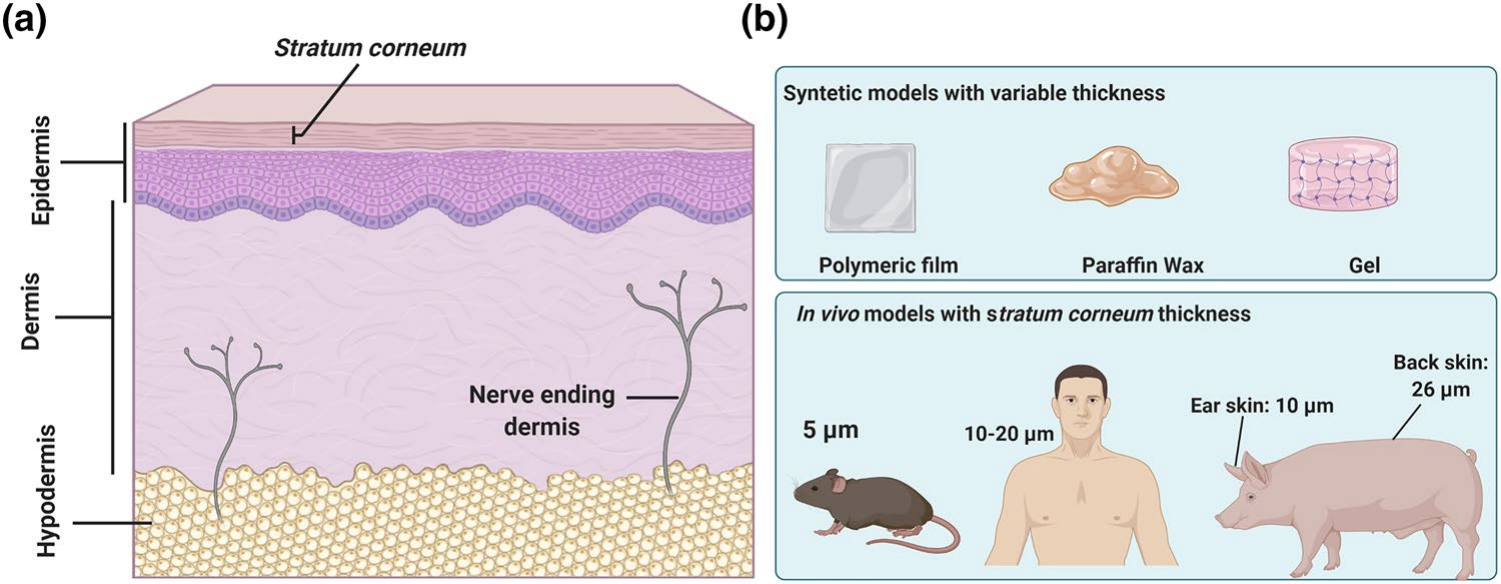
**a** Diagram on human skin and its layer. **b** Schematic illustration of *stratum corneum* thickness for in vitro and in vivo microneedle insertion

The human skin is most suitable for the evaluation of transdermal delivery; however, its use has been limited by ethical and laboratory approval as well as regulatory issues [90]. Hence, researchers have often used skin tissue samples derived from porcine and mouse subjects as an alternative for in vivo analysis of human skin (Fig. 7) [91–93]. Porcine skin is histologically similar to human skin, wherein the thickness of the *stratum corneum* of porcine back skin and porcine ear skin is 26 and 10 µm thick, respectively [94]. Furthermore, there is a close resemblance of epidermis thickness, dermal: epidermal layer ratio, density of hair follicles and blood vessels, dermal collagen: elastin ratio between the human and porcine skins [90]. If MNs are able to successfully insert in to porcine flank skin, it is assumed that they would be capable of inserting into human skin. This is not found to be the case, however, if insertion tests are performed on porcine ear skin, due to how thin this type of skin is compared to human skin [95]. The same issue occurs with insertion tests performed on mouse skin, which has a thinner *stratum corneum* (5 µm thick on the back) than human skin [95].

Apart from in vivo tests, commercial polymeric films such as Parafilm® M (PF), a blend of a hydrocarbon wax and polyolefin, may be used as a model membrane to analyze MN insertion depth, as a preliminary alternative to the use of biological tissues [96]. The artificial skin model overcomes the limitations associated with biological skin, including higher cost, the limited availability of fresh human skin samples, the difficulty of stretching skin to its in vivo configuration, and concerns regarding the safety of handling [90]. Several recent studies have used eight layers of PF as a skin simulant to evaluate the insertion properties of MNs [97, 98]. Skin penetration studies with polymeric films are typically performed before testing on human [93], mouse [91, 92] or porcine skin ex vivo [99]. As alternatives to animal skin models, paraffin wax and agarose gel can be used. Agarose is a carbohydrate polymer, generally extracted from certain red seaweed. Agarose gel is considered a suitable skin model because it can be designed with controlled mechanical properties to fit the human skin. Besides, the penetration depth over time is visible owing to the transparency of the material [100, 101].

Other gel-based polysaccharides, e.g., gelatin gels, are also employed as a skin model. Gelatin is generally derived from collagen taken from animal body parts [102]. To this aim, gelatin is melted and cured by UV light. Then, a thin layer of PDMS (~ 10 µm thickness) is covered on gelatin to simulate the *stratum corneum* layer of human skin. Such a strategy may be deployed to investigate drug release and needle penetration simultaneously [103].

## Skin Penetration Analysis

### Numerical Simulation

Finite element method (FEM) has recently become a powerful method utilized in simulations of engineering and mathematical models. For example, the analytical model of the skin, as a viscoelastic material [81], contains differential terms with so complicated analytical solution [104]. FEM software implements a particular numerical method to facilitate solving the partial differential equations governing the physical modeling. This tool allows engineers to investigate physical simulations without the need for experimental tests and therefore predicts the behavior of real systems. Some well-known softwares in this regard are ANSYS [105, 106], ABAQUS [107, 108], COMSOL [78, 109, 110, 111], and AutoFEM Lite [112]. Simulation softwares are used to predict the outcomes of drug delivery studies, skin penetration studies and the required structural analysis.

There are some general data necessary for the simulation to be representative of a drug permeation experiment involving MNs: geometry of the needle, material properties, boundary conditions and applied load. Mechanical properties of the skin provide a significant challenge for simulation software. Several models are used to stimulate the skin’s behavior. In this regard, hyperelastic models like Neo Hookian [107, 108] model and the linear elastic model [111, 113] may be implemented to simulate the skin behavior. Alternatively, simulations may consider the skin to be a mono-layer [108] or, more similarly to “real” skin, a multilayer [106, 107, 111, 113, 114]. By utilizing the multilayer skin model, layers can be simulated, which have different parameters within the same model.

The linear elastic model has been employed for MNs in almost all simulations [105, 106, 109, 111, 112]. In most studies using this model, the load is a force that applied to the needle array. This load can also be presented by displacement. In this regard, the needle’s penetration speed is constant and as small as the penetration can be considered quasi-static [106, 107]. It is worth noting that, in general, the penetration occurs when the substrate (Here, the substrate is skin) fractures. Usually, this failure is presented by an element deletion algorithm. Therefore, the element will be removed after satisfying a necessary condition [107].

One of the common applications of the FEM simulation is to investigate the effect of MN material on its efficiency, either for fabrication or for mechanical integrity. In light of this, Parker et al. [78] implemented a two-dimensional FEM simulation to investigate the buckling load, as mechanical performance, of titanium micromachining for the fabrication of MNs. In another study, 3D FEM simulations were employed to evaluate the ability of the fish-scale-derived microneedles to tolerate the force necessary to penetrate the skin, without fracture, compared to MNs fabricated with PMVE/MA [112].

**Fig. 8:**
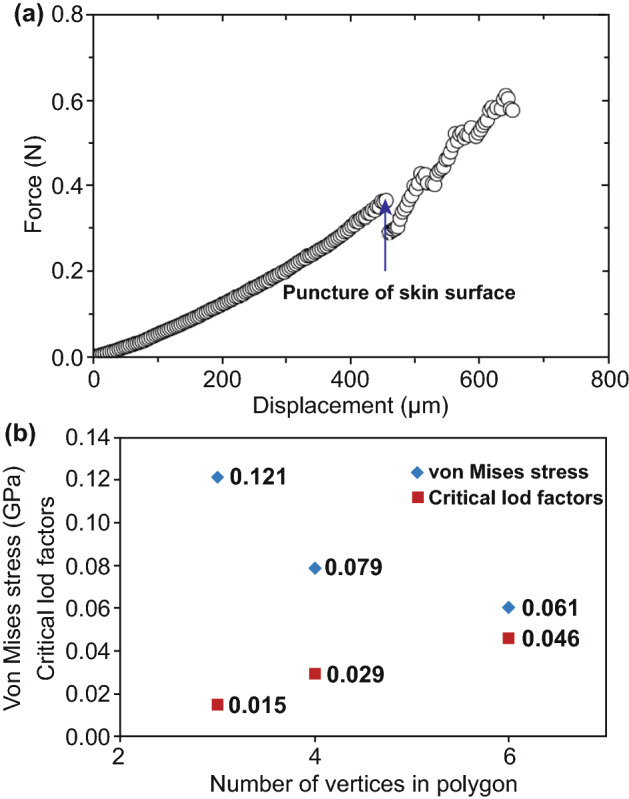
**a** General force–displacement behavior of a microneedle, plotted by FEM simulation. Reprinted from Ref. [107] with permission from Taylor and Francis. **b** Valuation of the triangle-, square-, and hexagon-based MNs based on their Von Mises stress and critical load factor. Reprinted from Ref. [111] with permission from Wiley

Investigating the shape and geometry of the MN is another application of the FEM simulation. For instance, the stress concentration areas and values for straight, jagged, and harpoon-shaped needles were studied with this method [105]. In another study, Kong et al. [107] studied the effects of geometry (i.e., tip area, wall angle, and wall thickness) on the deformation and failure of the skin and the insertion force. They presented the general force–displacement plot of MN’s skin penetration using FEM simulation (Fig. 8a). The sudden decrease in force refers to the point of needle insertion into the skin. In particular, effects of *stratum corneum* thickness, dermis thickness, hypodermis thickness, microneedle tip area, hollow microneedle wall angle, and hollow microneedle wall thickness on insertion force were investigated and related diagrams were plotted. These investigations concluded that tapered microneedles insertion can be optimized with *stratum corneum* properties, needle tip area, and needle wall angle. Besides, while increasing the wall thickness of hollow MNs with a large tip diameter enhances the insertion force, it has almost no effect on insertion force of hollow MNs with a small tip diameter. Also, Loizidou et al. [111] used FEM simulation to extract the Von Mises stress and critical load factor, as mechanical evaluation parameters, of MNs with triangle, square, and hexagon base geometries (Fig. 8b). The simulations indicated that by increasing the number of vertices in the polygon structure, MNs can withstand higher compressive loads. Accordingly, hexagonal-based MNs were found to be better able to withstand compressive loads than triangle-based MNs. Olatunji et al. [110] considered a general relationship for the insertion force as:

7.1 $$ F_{{{\text{insertion}}}} = F_{{{\text{bending}}}} + F_{{{\text{indentation}}}} + F_{{{\text{cutting}}}} + F_{{{\text{buckling}}}} + F_{{{\text{Friction}}}} $$

where $F_{{{\text{bending}}}}$ bends the skin, $F_{{{\text{indentation}}}}$ interrupts the SC layer, $F_{{{\text{cutting}}}}$ pierces the skin, $F_{{{\text{buckling}}}}$ deforms the skin and $F_{{{\text{Friction}}}}$ presents the frictional force during the penetration. Several analytical relations are developed for each of these forces. Then, the results of these analytical relations are compared with the results of a 2D finite element simulation. The authors concluded that both mechanical properties of the skin and geometry and alignment of the MNs on the patch affect the force components.

### In Vitro and In Vivo Models

In the past, skin penetration following MN application was confirmed by either applying a colored dye to the skin’s surface or by measuring TEWL after MN removal [34, 74, 115]. These techniques confirmed whether or not the *stratum corneum* had been breached; however, they provided no information on the needle penetration depth. To overcome this, various invasive and noninvasive techniques have now been developed to image MN skin indentation, with “true” depth results being demonstrated by optical coherence tomography (OCT) and confocal microscopy [114, 116, 117, 118].

Confocal laser scanning microscopy (CLSM) of skin cryo-sections may be used to investigate the penetration of MNs through the skin layers [91, 119], in addition to the permeation of naturally fluorescent or fluorescently tagged drugs. By producing high-resolution images, CLSM allows one to measure the dimensions of the holes created by the MN and the closure of such conduits over time [120]. For example, one study visualized the 250-µm moon-shaped conduits left in dermatomed human skin following MN application [121]. Apart from confirming the insertion of MN arrays, CLSM can also be used to show whether the MNs can reach the viable epidermal and dermal layers [122]. This enables the formulator to prove that the MNs can indeed deliver their cargo to the vascularized regions of the skin for efficient absorption into the systemic circulation.

MN characterization and the presence of the composite within the MN formulation have been imaged using CLSM. For example, one study characterized “fast-separable” MNs for nanoparticle delivery using CLSM [123]. This study used CLSM to indicate the empty holes present in skin following MN insertion, though the CLSM image initially showed no nanoparticle release, indicating that the MN formulation required optimization. After introducing an external source of fluid to induce rapid MN dissolution, the same study used CLSM to indicate the successful release of the nanoparticle composite, thus illustrating that the optimized MN formulation was able to release the desired nanoparticle load into the skin [123].

OCT is a noninvasive and high-quality imaging method that provides cross-sectional images from a light-scattering media such as biological tissues. Abnormalities recognized in OCT imaging are beneficial for the diagnosis of various diseases such as neurological disorders [124]. This imaging technique is based on low-coherence interferometry and according to the reflected light intensity creates pseudo-color images of the tissue [24]. Appreciating the benefits of OCT imaging and the wealth of information this can provide, formulators have now employed this technique for MN development. Using OCT imaging, MN insertion can be imaged in real time following application to both artificial and biological matrices. Therefore, using this technique, one can decide early on in the development stage whether the MN possesses the required characteristics for effective skin penetration.

**Fig. 9:**
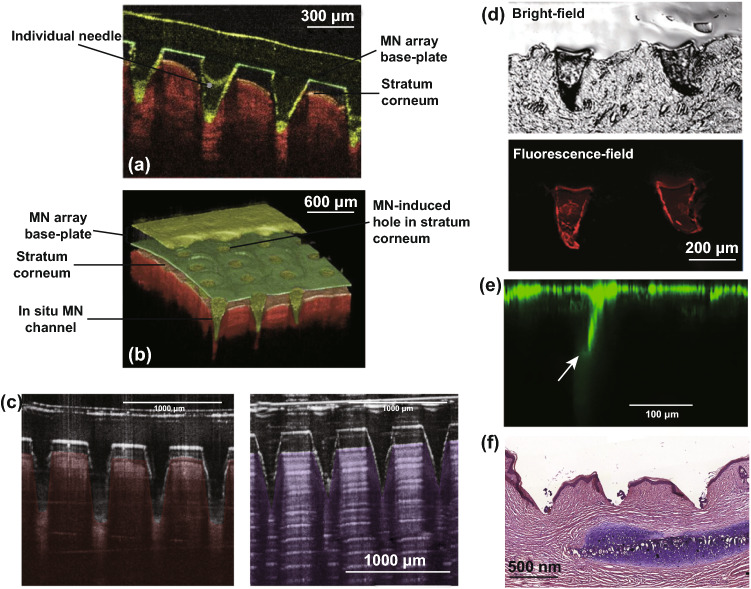
**a** Optical coherence tomography (OCT) 2D cross-sectional image from penetration of polymeric MNs arrays with height of 600 µm into human forearm skin. **b** 3D OCT image created from 2D images by Ability Photopaint®. Reprinted from Ref. [125] with permission from Springer. **c** Optical coherence tomography images taken from MNs inserted in neonatal pig skin (left panel) and eight layers of Parafilm® M (right panel). Reprinted from Ref. [96] with permission from Elsevier. **d** Bright-field and fluorescence-field images of arrowheads inserted into mouse skin. Reprinted from Ref. [126] with permission from Royal Society of Chemistry. **e** Visualization of the transport of 200-nm FITC-nanoparticles into human skin (up to 250 μm) after using confocal microscopy. The conduit is indicated by an arrow. Reprinted from Ref. [121] with permission from Elsevier. **f** Images of hematoxylin and eosin (H&E)-stained section of hypertrophic scar tissue (7 µm) after the application of MNs. Reprinted from Ref. [85] with permission from Elsevier

Another valuable property of OCT is the capability of taking 2D and 3D digital images (Fig. 9a, b). The penetration of polymeric MNs in the forearm skin of volunteers has been studied by VivoSight® high-resolution OCT Scanner with a handheld probe. To enhance real-time high-resolution imaging, a laser center wavelength of almost 1305 nm was embedded in the swept-source Fourier domain OCT system. 2D images were then transformed to 3D using an imaging software (ImageJ®), and a false coloration was added to the images to differentiate between MNs and skin layers (Ability Photopaint®) [28].

OCT continues to be an established method for assessing MN insertion. For example, OCT was recently used to image the insertion of MN arrays manufactured using a two-photon polymerization technique [127]. In this study, OCT was used to measure the percentage of needles inserted into PF, an established model membrane used for MN insertion studies as discussed previously [127]. Furthermore, successful MN insertion into full-thickness neonatal porcine skin was also illustrated using OCT.

Highlighting the applications of OCT imaging for MN development, Larraneta et al. directly compared MN indentation in an artificial and a biological membrane, namely 8 layers of PF and excised full-thickness neonatal porcine skin, as shown in Fig. 9c, d [87, 128]. Interestingly, the penetration depth in both the artificial and biological membranes was directly comparable following the application of a uniform force [128]. As a result, the authors proposed the use of PF in conjunction with OCT imaging as a possible quality control test for MN manufacture.

Bright-field microscopy can also be employed to evaluate MN indentation. This is one of the simplest forms of optical microscopy in which light is absorbed by dense areas within the sample (Fig. 9e) [129]. This generates a contrast within the sample, which in this example enables the needle and upper layers of the skin to be distinguished. Although bright-field microscopy is classified as a standard light microscopy technique, its use is limited due to low optical resolution at higher magnifications and poor contrast with most biological materials [130]. Fluorescence-field microscopy can be useful for evaluating the insertion ability of the MNs ex vivo [126]. This imaging technique provides a contrast between the needle tips loaded with a fluorescent dye and the viable skin layers. In addition, fluorescence microscopy can be used to visualize the diffusion of fluorescent compounds within the viable skin layers following successful MN indentation [126]. Figure 9f illustrates an optical microscopy image from a skin cross-section after MNs removal. The stained piece of skin under ×10 magnification shows the depth of the holes created by the MNs [85]. Rhodamine B dye was used to reveal drug distribution within the needles, and MN holes were dyed using trypan blue solution. Methylene blue is also another common dye used for demonstrating successful MN insertion [131, 132]. These dyes are used as they are able to stain viable cells, such as those of the epidermis, but not the “dead” *stratum corneum*, and thus, MN penetration depth can be accurately measured.

## Parameters Affecting MN Insertion

There are numerous different factors that affect skin insertion of MNs. This section discusses the parameters (e.g., the geometry of the needles, applied materials, amount of filler and cross-linking agents) that influence skin penetration.

### Geometry

**Fig. 10:**
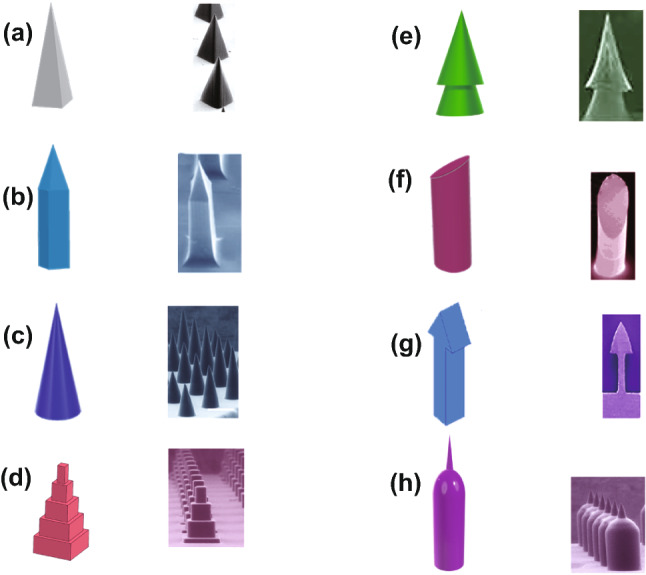
Different microneedle designs and their constructed needles. The SEM images of part **a** [135], **b** [136], **c** [135], **d** [137], **e** [138], **f** [139], **g** [140] and **h** [135] reprinted with permission

Skin is inherently elastic, and because of this, MNs must have a geometry which is able to overcome skin’s elasticity for adequate penetration. The geometry of MN arrays (including the space between needles, needle shape, needle tip diameter, and base diameter) must be carefully considered to enable that they can penetrate the skin successfully and in a relatively painless manner. Needles should also have an optimal height to pass the *stratum corneum* and also to avoid pain generated by nerve contact. Numerous studies have investigated the effects of MN geometry on effective skin insertion [19, 22, 117, 133, 134]. Within these studies, trends have emerged which provide an insight into the most important factors that affect MN insertion. For example, one study found that increasing the interfacial area of the MN tip increased the insertion forces required, and increasing tip radius, wall angle and wall thickness increased the fracture force [19]. This result was shared with a study that explored the effect of MN geometry on skin permeability [22]. The study found that increasing the width, length and decreasing the space between needles lead to greater effective skin permeability. This is understandable, given that needles of increasing width, length and more densely packed MNs lead to wider, longer and more densely packed holes, through which a greater amount of drug can diffuse through. However, more densely packed needles may lead to the “bed-of-nails” effect. Furthermore, increasing the needle tip radius may decrease the likelihood of sufficient needle insertion due to the wider contact angle, and it may also increase the risk of pain due to the increased potential for nerve contact. Figure 10 represents different types of geometry used for MNs.

Computerized tomography (CT) scanning has been used to evaluate the effects of MN geometry on skin penetration [111]. In this study, laser micromachining was used to prepare MNs of varying shapes (triangular, square and hexagonal), whilst keeping the space between needles, base diameter and MN length the same. The ex vivo penetration characteristics of each array type were investigated using CT-scan imaging. The results indicated that increasing the number of vertices (hexagonal > square > triangular) improved the mechanical properties of the needles. However, the increasing the number of vertices decreased the ability of the needles to penetration the skin. Simulation software predicted that hexagonal MNs would be able to withstand higher critical buckling loads and compressive stress than square or triangular MNs and triangular MNs showed enhanced skin penetration compared to hexagonal MNs. The greater ability to insert into skin was attributed to the sharper edges of the triangular and square MNs compared to the hexagonal MNs.

The effects of MN geometry (needle type, density, shape and length) on transdermal delivery of zolmitriptan have recently been investigated [141]. Various lengths of AdminPatch® (SS316L stainless steel) were tested (600, 900, 1200 and 1500 µm), and with increasing needle length, increasing permeation of zolmitriptan was indicated. This is to be expected, as increasing needle length increases the depth of drug delivery, and the deeper the drug delivery is, the more likely the drug will be delivered into the dermal layer and thus will be available for uptake by the dermal microcirculation. Of the two types of MNs used (AdminPatch® and laboratory-fabricated polymeric MNs, both 600 µm), the polymeric MNs formed the wider and deeper microconduits, thought to be due to their conical shape. This resulted in a 3.6-fold increase in zolmitriptan delivery from the polymeric MN arrays compared to passive delivery, and the AdminPatch® delivered 3.17-fold more zolmitriptan compared to passive delivery. However, the polymeric MNs were applied thrice to maintain a MN density closer to that of the AdminPatch®. While this allows a more direct comparison between the two patch types, a triple application of the polymeric MNs may have contributed to the increase in zolmitriptan delivery when compared to the AdminPatch®.

In addition to drug delivery, MNs have been used for transcutaneous immunization and the effects of MN geometries on the feasibility of transcutaneous immunization have been investigated [142]. In one study, the geometry of dissolving MNs was investigated for the delivery of ovalbumin [143]. The results of this study indicated that the needle morphology of dissolving MNs influenced their mechanical properties, dissolution and insertion capacity, which in turn affected the immune response. In this study, cone-shaped MNs were found to be the optimal shape for ovalbumin delivery and transcutaneous immunization [143]. This was due to the relationship between apex angles and length-diameter ratios—cone-cylinder MNs used in this study had an increasing apex angle with a decreasing diameter, which increased the risk of needle fracture when force was applied. The opposite was found to be the case for the optimal cone-shaped, dissolving MNs. Furthermore, cone-shaped MNs were found to have the fastest dissolution time. Therefore, greater needle insertion and a fast dissolution time resulted in greater ovalbumin delivery and a more potent immune response.

**Fig. 11:**
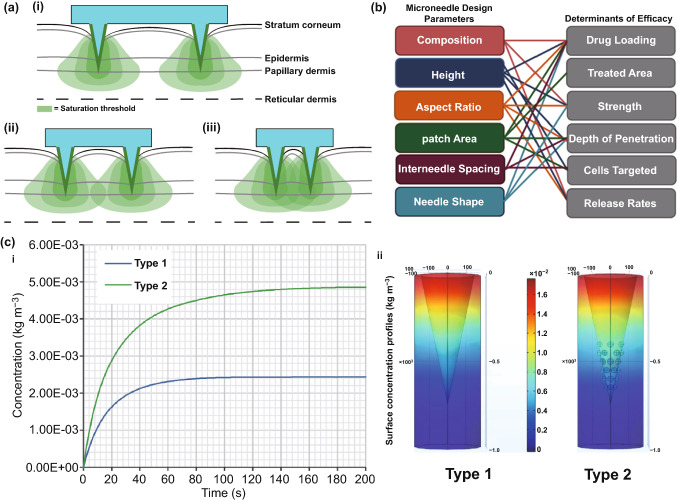
**a** The number of cells activated by delivery of a dermal vaccine was dependent on MN spacing, as shown in the 2D drawings of a large (i), optimal (ii), and small (iii) distance between MNs. Reprinted from Ref. [145] with permission from Taylor and Francis. **b** Relationship between microneedle design parameters and therapeutic efficacy. Reprinted from Ref. [135] with permission from Plos One. **c** Time concentration profile (i) of a standard conical dissolving MN shape (type 1) and a novel shape with hemispherical convexities to maximize drug delivery when partial needle insertion occurs (type 2) with surface concentration profiles (ii). Reprinted from Ref. [18] with permission from Elsevier

With the increasing growth in the transdermal market [144] and the push toward clinical application of MNs, it is important to create reproducible tests for regulatory acceptance criteria. Though ex vivo skin is the closest surrogate to human skin and is thus used in numerous experiments investigating drug delivery using MNs, such a skin model cannot be used to reproducibly evaluate MN insertion. PF has been found to be of use for quality control testing of MN insertion as described previously [128]. An alternative to this method is the use of a computational model, which may be able to predict the optimal geometry of MNs for skin insertion [145]. Such a computational model has been used to predict the optimal MN geometry, specifically for dermal vaccination [145]. This study investigated the effect of spacing between MNs, MN length and MN array base radius on needle insertion and immune response. The results revealed that the optimum distance between MNs was influenced by the quantity of activated antigen presenting cells and the target site (epidermis or dermis), which was thought to be related to the immune response induced by the antigen-presenting cells (Fig. 11a). The maximum number of activated antigen-presenting cells occurred when the spacing between individual MNs was at a distance of 1 mm and 1.5 mm when targeting the epidermis and dermis, respectively. In addition, it was found that the MN length affected the quantity of antigen-presenting cells that were activated, with increasing MN lengths suited to dermal antigen-presenting cell activation. The array base radius had minimal influence on the number of immune cells that responded. Figure 11b represents the relationship between MN design parameters and therapeutic efficacy.

More recently, a computational model was used to assess the potential of a novel dissolving MN array design for drug delivery applications [18]. A simulation of the MN dissolution process in a porous medium was executed, which was validated using a study of dissolving MNs for drug delivery to the cornea. From the obtained results, a new MN shape was proposed, consisting of a cone shape with an array of hemispherical convexities located in the lower half of the MN, to reduce the risk of delivery of an insufficient amount of drug if partial needle penetration occurred, reducing drug waste, whilst increasing drug flux (Fig. 11c).

The relationship between MN geometry and force required to insert MNs has also been investigated [146]. The insertion force was found to strongly relate to tip angle and the radius of the MN tips curvature. The insertion force increased with increasing width of MN shaft, but the relation was weak, and the radius was found to have no influence on the insertion force.

In relation to the needle density, a phenomenon known as the “bed-of-nails” effect may occur. Whilst increasing the number of needles cm^−2^ on a single baseplate appears useful for increasing the amount of drug deliverable using MN arrays, applying an equal application force to a MN array of higher density may result in a lower force applied to each individual MN, resulting in reduced needle insertion and thus reduced drug delivery. This phenomenon has been demonstrated numerous times within the literature [147, 148]. For example, human epidermis pretreated with MNs (400 µm needle length and 2000 needles cm^−2^ density) resulted in a significant enhancement in aciclovir flux; however, when using MNs with the same length but with an increased needle density (5625 needle cm^−2^), a lower enhancement of drug flux occurred [149].

The “bed-of-nails” effect has been explained more recently using the finite element analysis of a 3 × 3 hafnium oxide MN array [150]. Reducing the spacing between individual MNs (from 80 to 20 µm) resulted in smaller MN maximal height difference (i.e., the difference in MN height after application to the skin when compared to the original MN height before application to the skin), which caused a reduced maximal stress in skin. Thus, when the same pressure was applied to the MNs of different spacing, the skin was not pierced when the MN spacing decreased to 20 µm [150].

It is clear from the studies summarized in this review that the geometry of MNs can be altered to optimize the insertion of MNs into skin. It is important to note that optimization of MNs for skin insertion cannot be considered a “one-size-fits-all” approach and must be considered alongside factors such as skin elasticity and, in particular, the transdermal target, as can be seen from the Römgens et al.’s study [146]. Furthermore, there is a balance that must be struck between effective skin insertion and skin permeability. Though MNs exhibiting a greater length, width, and needles with a greater density are thought to lead to greater effective skin permeability [22], outliers exist in the form of the “bed-of-nails” effect, which may reduce skin permeability despite an increase in needle number within a single array, as described above. Furthermore, skin permeability is likely to be increased by the length of needles; however, increasing MN length increases the risk of nerve contact, resulting in pain upon application, which may reduce patient acceptance of the device, an important aspect to consider when developing MNs for clinical applications.

### Microneedle Materials

In addition to the geometry of MNs, the material used to fabricate MNs can have a profound effect on its ability to sufficiently pierce the skin. Whilst the ability to insert efficiently may be considered one of the most important factors when fabricating MNs, other factors must be considered alongside this. Such factors include cost, biocompatibility and batch production capabilities, and therefore, MNs that readily insert into the skin cannot necessarily be considered viable for clinical applications due to the combination of these other factors.

The first material used for MN fabrication was silicon, and this material has a high flexibility in the processes that are used to form the material. This has allowed a wealth of MN sizes and shapes to be manufactured, which has already been discussed as having an influence on MN insertion. Silicon MNs have significant strength mechanically, and this allows silicon MNs to sufficiently insert into skin [151, 152]. Deformation and stress testing of silicon MNs of concave conic shape were undertaken to predict the incidence of MN deformation [153]. A skin penetration study was conducted on rat skin, where it was found that with increasing load (50–800 g), surface buckling deformation did not occur, indicating a good mechanical strength of the needles. Previously, there have been concerns surrounding silicon biocompatibility due to the possibility of needle fracture into the skin, as although silicon MNs are mechanically strong, they are also brittle [154]. However, more recently, researchers have character silicon MNs for drug delivery applications [155]. The needles passed the Vickers hardness test, and thus, the authors believed that these needles would be easily inserted into skin for drug delivery applications, without the possibility of needle fracture.

Silica glass is an alternative material that has also been investigated for MN fabrication. Borosilicate glass in particular presents lower elastic moduli values (64 Gpa) than silicon [156]. However, as indicated in Fig. 6, silica glass and silicon have similar fracture toughness values, and this, combined with the time inefficiencies associated with silica glass fabrication [139], means that glass MNs cannot realistically be used commercially and are instead reserved for experimental purposes only [157].

Metals are another common material used to fabricate MNs. Metals such as titanium and stainless steel have already been used for hypodermic needle fabrication, medical implants and prostheses, and have shown good biocompatibility and mechanical strength. Young’s moduli measures the elasticity of a material. Metals may be considered comparable to the highest possible value of silicon (180 GPa) when comparing moduli values; however, metals exhibit a higher fracture toughness compared to silicon. Thus, metals could be considered a more suitable material for MN fabrication compared to silicon [87]. Table 1 represents the strengths of materials used in microneedle fabrication.

**Table 1:** Strengths of materials used in microneedle fabrication

Material	Young’s modulus (GPa)	Ultimate tensile strength (MPa)
Silicon	110	7000
Glass	85	50
Nickel	214	586
Palladium	117	186
Platinum	147	117
Titanium	110	241
Stainless steel	200	1000
Ormocer®	17	30
Polymethyl methacrylate	3	170
Poly(glycolic acid)	12.5	890 [158]
PLGA 75/25	629 [159]	28 [159]
PLGA/PCL/PLGA (25/50/25)	26 [159]	2 [159]
PLGA/PCL/PLGA (37.5/25/37.5)	20 [159]	2 [159]
Maltose	31	–
Hyaluronic acid	2 × 10^−4^ [160]	20 [160]
Poly(d,l-lactide)	2.7 [161]	128 [162]

Data obtained from [163] except those mentioned

Ormocer®: “Organically Modified Ceramic Technology,” containing inorganic–organic co-polymers in addition to the inorganic silanated filler particles [164]

Alumina (Al_2_O_3_) is the most popular type of ceramic for the production of MNs [165–167]. When compared to monocrystalline silicon, alumina has a suitable mechanical strength (fracture toughness of 3.75–4.85 and 0.83·–0.94 MPa m^1/2^) [168]. Though alumina is considered to be one of the most stable oxides and thus is not affected by adverse environmental conditions, or corrosion [169], it is a brittle material (Fig. 6a) and has a lower strength resistance to tension when compared to other materials (Table 1). Its brittleness has been demonstrated in mechanical performance tests using microindentation techniques eliminating shear forces and by manual application of MNs into silicone rubber. After manual application of the ceramic MNs, some MNs were broken and left in the silicone rubber [165] and are therefore unsuitable for insertion into human skin. Zirconia is another type of ceramic, which has a better bending strength and fracture toughness values compared and bending strength when compared to alumina, which may be more attractive from a MN insertion standpoint, but it has poorer wear characteristics [170].

Despite the apparent disadvantages of ceramics for MN insertion and thus transdermal drug delivery applications, they offer controlled and adjustable porosity, which can be taken advantage of to enhance percutaneous drug permeation [171]. The drug is either loaded into the MN pores and is able to diffuse out of the pores as a liquid formulation once the array pierces the skin and the array comes into contact with interstitial fluid, or a drug formulation is loaded into MN pores and subsequently dried. Once the needles pierce into the skin, the formulation is hydrated with interstitial fluid and the drug is transported from the MN pores into the skin [168]. This second technique was used to deliver ovalbumin to skin dendritic cells using MNs, i.e., biocompatible ceramic MNs [172]. These MNs have the advantages of ceramic MNs without the problems associated with polymeric MNs, such as their low mechanical strength (and thus poor skin insertion) and their inability to withstand high temperatures during the fabrication process [87, 173, 174]. However, insertion studies using ex vivo* human* skin indicated that 36.4% and 27.5% of 300 and 600 µm length needles were broken, respectively. The authors did not specify whether the needle fragments were found within the ex vivo skin, but nonetheless, it appears as though ceramic MNs are a poor choice for clinical applications due to their brittle nature [172].

Carbohydrates are cheap and safe for human health and thus have also been used to fabricate MNs [175]. Some studies have indicated that such “sugar MNs” are able to penetrate the skin [116, 176]—but even in these cases, the needles failed to penetrate near to the depth of the needles themselves (i.e., needle length of 508 µm but penetration depth of 160 µm). Whilst this could be attributed somewhat to skin elasticity, the lack of depth penetration is excessive, and thus, a lack of mechanical strength should also be considered. More recently, a study characterized structural properties of sugar MNs [109]. Results indicated that carboxymethylcellulose/maltose MNs exhibited better mechanical strength values when compared to carboxymethylcellulose/trehalose and carboxymethylcellulose/sucrose MNs. Buckling was the main mode of MN failure (in respect to transdermal propanol delivery), and there was a positive relationship between the order of buckling and Young’s modulus values of the sugar components of each MN (Table 1). These mechanical issues combined with other disadvantages associated with carbohydrate-based MNs [177] are likely to impede their commercial development.

Polymers are widely considered as the most popular material for MN fabrication, due to their suitable, biodegradability, biocompatibility, strength/toughness values, low toxicity and low cost [178, 179]. Polymers are used mainly for the fabrication of dissolving and hydrogel-forming MNs due to their high biocompatibility, reducing concerns surrounding potential MN material that may be left within the skin following insertion. Generally, polymers exhibit greater toughness than ceramics or glass, but poorer strength than metals, silicon, ceramic and glass (Table 1; Fig. 6c) [87]. Fortunately, different blends of polymers can be used to achieve the desired mechanical properties for a specific clinical application [81, 180]. If some polymers have insufficient piercing abilities, piercing materials can be used in conjunction with the MNs themselves. For example, one study used hyaluronic acid, carboxymethyl cellulose and alginate as a piercing material to pierce the skin quickly (through quick dissolution) and evenly, thereby allowing underlying MNs to interact with interstitial fluid [181]. This study found that a sharp needle tip and overall mechanical strength were the two most important factors affecting needle insertion. The most successful piercing material was hyaluronic acid at a concentration of 3% w/w (tip size of approximately 48 µm, dissolution time 1 min 30 s).

### Effect of Filler and Cross-linking

**Fig. 12:**
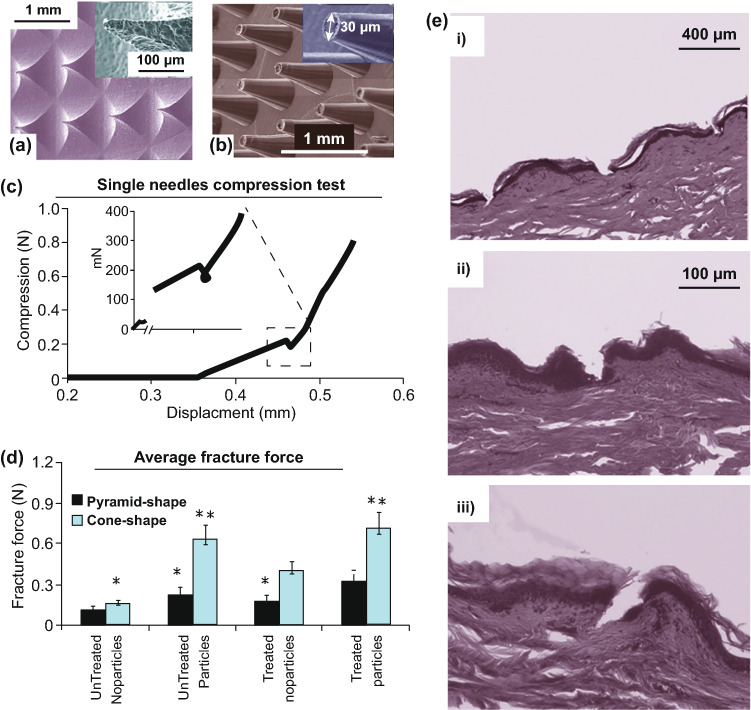
SEM image of the pyramid (**a**) and cone-shaped MNs (**b**). **c** A single-needle compression test that breaks around 225 mN during compression. **d** Fracture force of all subgroups of the pyramid and the cone-shaped MN fabricated by micromolding. **e** Hematoxylin and eosin images of human cadaver skin treated with cone-shaped silk MN: (i) MN loaded with microparticles and no post-treatment, (ii) MN with no microparticles and post-treatment with water vapor and (iii) MN loaded with microparticles and post-treated with water vapor. Reprinted from Ref. [184] with permission from Wiley

Whilst there is a large volume of literature which focused on the effects of composite on drug delivery, there is little regarding the effects of composite on needle insertion. It is worth noting that the mechanical strength of MNs may be weakened by the presence of drug within the MN matrix because drugs are mechanically weaker than polymers [182]. On the contrary, incorporating fillers such as metallic and polymeric particles in the matrix increase the elastic modulus of the hybrid polymer [91, 183]. In light of this, one study focused on the use of silk MNs, and more specifically, loading needles with silk microparticles to explore how this affected needle mechanical strength (Fig. 12) [184]. Incorporation of microparticles was found to increase mechanical strength of the needles. Pyramid-shaped MNs had a mean fracture force of 175 mN/needle, whereas the addition of microparticles increased average fracture force to 330 mN/needle. Mixing beta-sheet-induced microparticles with a silk solution formed a silk composite. This novel composite formulation reinforced the bulk silk material and was found to improve the mechanical stability of the resultant MNs. Cone-shaped MNs were also found to be superior in mechanical strength compared to pyramid-shaped MNs, again illustrating that MN geometry has an effect on mechanical strength [184].

Another study found that the incorporation of layered double hydroxide (LDH) nanoparticles within sodium carboxymethylcellulose (CMC) MNs formed a nanocomposite, which increased the mechanical strength of the needles without sacrificing dissolution rate [185]. The addition of 5% LDH nanoparticles was found to increase the mechanical strength of needles most significantly, which was measured by load–displacement, elastic modulus and hardness. For example, much greater loads were required for needle penetration of the same depth with increasing LDH concentration. The elastic modulus of pure CMC was 0.993 GPa, whereas the elastic modulus of 5% LDH-loaded CMC MNs was 2.878 GPa. The hardness of pure CMC polymer was 0.067 GPa; the hardness when 5% LDH was incorporated was 0.111 GPa [185].

Alternatively, one study found similar insertion profiles from dissolving MNs loaded with different concentrations of a rilpivirine nanosuspension, using the previously established PF method [128]. Four formulations were tested, and all were capable of piercing three layers of PF (approximate depth of 378 µm). MNs containing no PVA (100% rilpivirine) did not provide sufficient mechanical strength, despite being able to penetrate through PF, as some needles broke away from the array and were found lodged in the PF® [186]. This study indicates that altering the concentration of the composite may not necessarily change the mechanical strength of MNs; however, the presence of the composite may increase the needle mechanical strength. Thus, more studies are required to investigate this effect in detail. It is likely that such an effect may depend on the initial material used to formulate the MNS and may differ on a case-by-case basis.

**Fig. 13:**
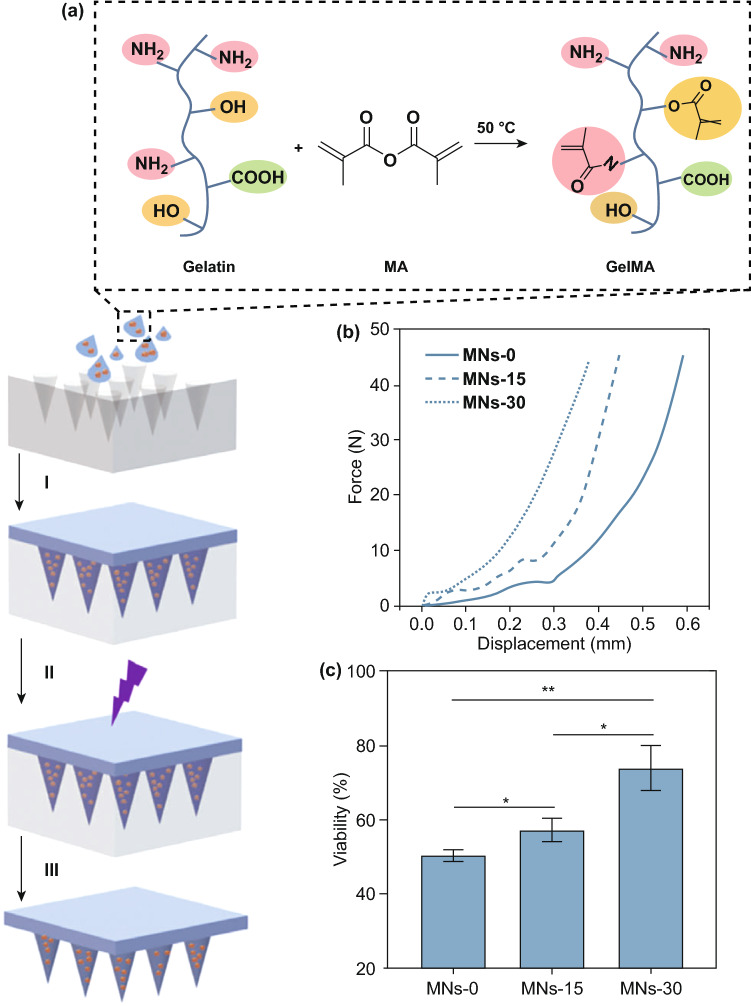
**a** Schematic of MN arrays fabrication process by (I) centrifugation, (II) UV cross-linking, (III) overnight dry and peeled off from PDMS mold. Effect of the duration of cross-linking on the mechanical strength (**b**) and biocompatibility **c** of the microneedle patches based on gelatin methacryloyl (GelMa). The MNs that cross-linked for 30 s exhibited the highest cell viability, whereas MNs without cross-linking or those cross-linked in 15 s showed the lower cell viability. Reprinted from Ref. [188] with permission from Wiley

The extent of cross-linking is a critical parameter associated with hydrogel-forming MNs. MN patches with greater cross-linking density need more force to cause the same amount of compression, signifying that enhancing cross-linking time considerably improves the mechanical strength of MNs [187]. Accordingly, systems with greater cross-linking density need more force to cause the same amount of compression (Fig. 13) [188].

A general rule is that the greater the cross-linking effect, the less able the array is to swell, and therefore, this generates smaller gaps through which a drug can diffuse into the interstitial fluid and eventually the dermal microcirculation. This is not always the case, and the size of the drug and potential interactions between the drug and MN material must always be considered. Thus, it is clear that the cross-linker has a great effect on the ability of a drug to diffuse through the MN material and into the skin. However, there has been little research into the effects of the cross-linker on MN insertion. It appears as though different cross-linking times have no effect on insertion capabilities [189]. Moreover, swelling and super-swelling MNs have been shown to have the same insertion depth, despite using different cross-linking methods [190]. It appears, therefore, that cross-linking does not have an effect on MN insertion, though it is nonetheless important to consider for drug delivery. However, the effect of cross-linking on MN insertion was not the main focus for either of these cited studies and should be studied in greater depth in the future.

## Strategies to Enhance MN Insertion

Since MNs were first conceptualized as a drug delivery device in 1971 [191], the technology continues to grow and more sophisticated and complex types of MNs have been developed to overcome the many challenges associated with transdermal drug delivery, whilst aiming to create a simple and convenient device that the end-user would be happy to use.

**Fig. 14:**
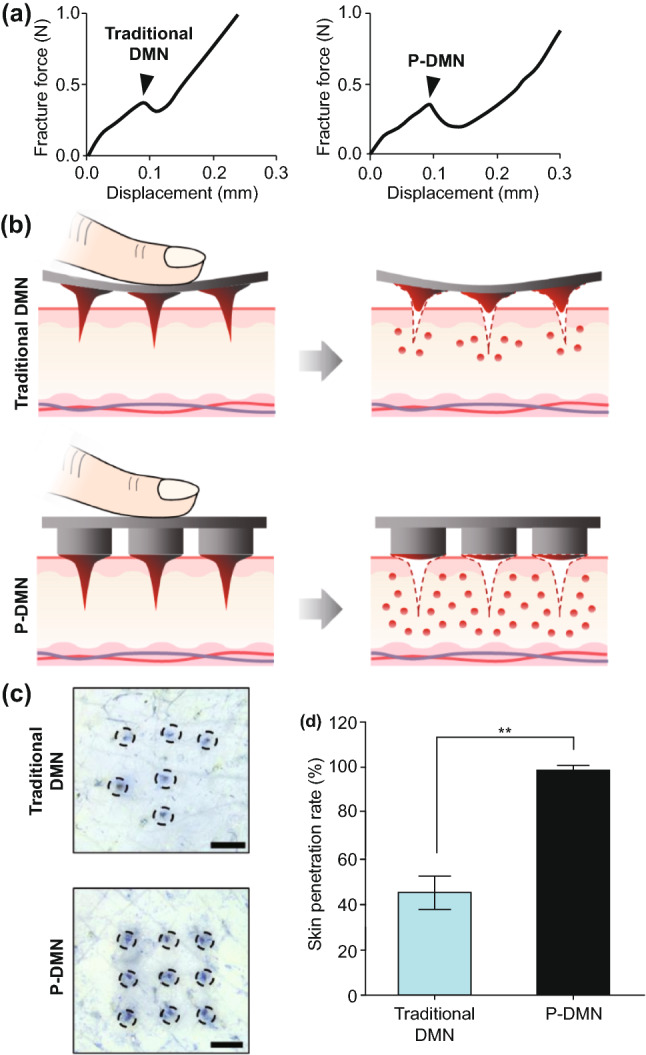
Evaluation of mechanical force and insertion of traditional MNs and pillar-integrated microneedles (P-DMNs). **a** The mechanical fracture force of DMNs proved their ability to puncture the skin without breakage. **b** Illustration of DMN application onto the skin. The uniform distribution of the application force in P-DMNs leads to complete delivery of the encapsulated drugs. **c** Traditional DMNs exhibited an incomplete array of spots, while a uniform pattern of 3 × 3 spots was achieved in P-DMN-treated skin. **d** Assessment of skin penetration. Reprinted from Ref. [192] with permission from MDPI

The large number of studies cited in this review demonstrates that due to the physicochemical properties of the skin, polymeric MNs may be unable to completely insert into the skin, leading to inconsistent and random delivery of drug cargo within MNs [192]. In the recent decade, significant progress has been made to ameliorate the insertion capability and efficiency of MNs. In some cases, the mechanical fracture force proved that MNs have the desired mechanical strength to perforate the skin without breakage, but they are unable to successfully insert. The delivery efficiency of MNs depends on many different factors; for instance, application force and skin elasticity. If the skin is too elastic, MNs tend to push the skin instead of penetrating and creating holes to deliver the drug cargo. Fabricating MNs over micropillars makes it possible to distribute the force over each MN and perform a uniform and complete insertion of the whole array into the skin (Fig. 14) [192].

Pedestal-based MNs (also known as pillar integrated MNs) have an extended length to counteract the elasticity of the skin and provide additional mechanical strength to aid in successful penetration/insertion [193–197]. Taking this into account, pillars could be fabricated by a dissolvable polymer, e.g., PVP or PVA, which dissolve immediately after insertion of MNs into the skin. In some other cases, polymers like PCL and PLA could be utilized as a separatable strong base which eventually separate from the MNs upon insertion.

Insertion-responsive MNs (IRMNs) rely upon the strength of adhesion between materials for tip separation, rather than mechanical interlocking. When IRMNs insert into the skin, mechanical stress is exerted, thereby inducing cracking and subsequent separation at the tip–base interface. Upon removal, the inserted tips are separated from the base and the coated drug or vaccine is released into the skin. IRMNs were developed to allow for skin insertion without the need for a patch.

**Fig. 15:**
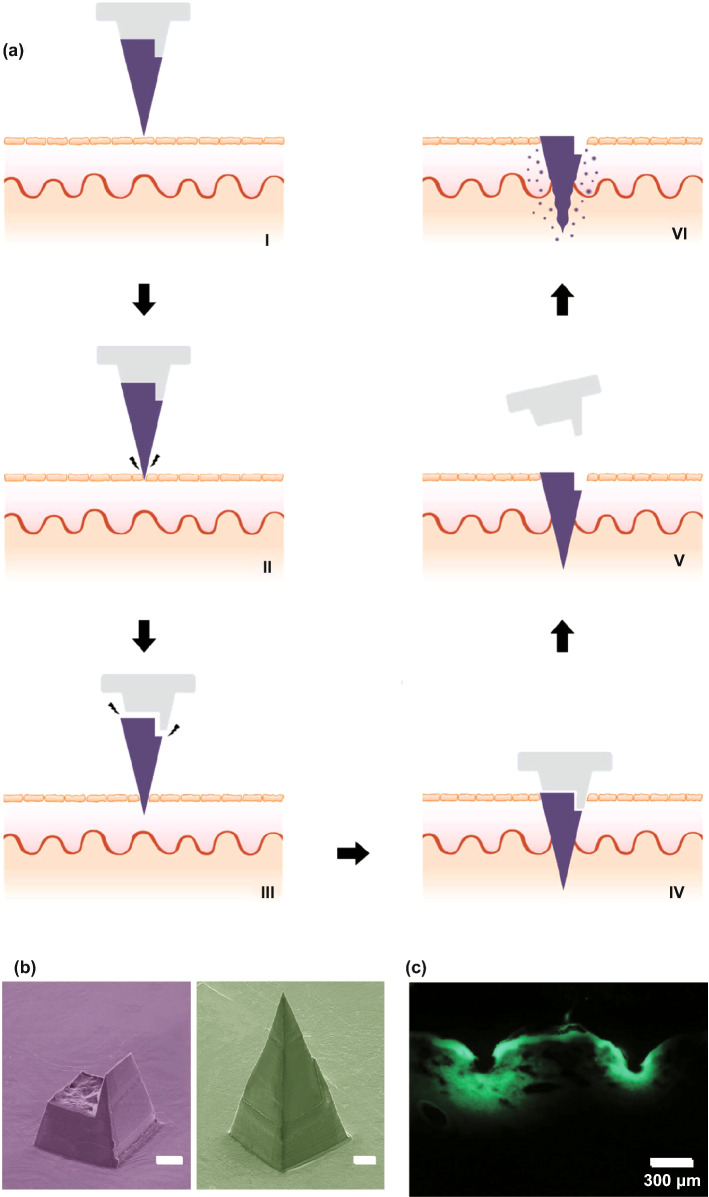
**a**I–**a**VI Insertion-responsive MNs, which use two different materials to form the MN base and MN tips. Upon skin insertion, separation of the tip from the rest of the MN occurs immediately and the tip remains embedded within skin tissue, and the rest of the MN is removed from the skin, allowing the inserted tip to slowly release the drug [197]. **b** SEM images of an a single-walled stand (left), and an insertion-responsive MN with a single-walled stand (right). Scale bar = 100 µm. **c** Cross sections of porcine skin following insertion-responsive MN insertion. MNs were loaded with fluorescein as a model drug. Scale bar = 300 µm. Reprinted from Ref. [197] with permission from Royal Society of Chemistry

One study investigated the effect of base geometry (with or without a walled square pyramid stand) on the mechanical behavior of IRMNs (Fig. 15) [197]. Pyramidal and square hyaluronic acid MN tips with a polycaprolactone base were manufactured. Following skin insertion, hyaluronic acid tips were separated from the polycaprolactone base due to the relatively weak adhesion strength between polycaprolactone base and hyaluronic acid. Ex vivo skin insertion tests confirmed that separation of the tips from the base array occurred following insertion, irrespective of the presence of a wall on the stand. However, only IRMNs with the single-walled square pyramid stand were deeply embedded within the skin. Mechanical testing results illustrated that the when a wall was present on the base, the mechanical stability of the IRMNs was increased. The presence of the wall also allowed for suitable adhesion between the tips and base, preventing tips breakage during insertion, whilst still allowing the needle tip to separate from the baseplate upon removal [197].

In two studies, such IRMNs were used to deliver canine influenza vaccine [198, 199]. Canine influenza vaccine was coated onto the tips of water-soluble IRMNs and delivered immediately following IRMN insertion into skin. Skin insertion tests were conducted using full-thickness porcine skin with fluorescein staining. The needle arrays inserted into skin for 10 s; it was observed that 100% of the tips were successfully separated from the baseplate and all tips remained inserted in the skin following baseplate removal. However, due to skin elasticity, the MN tips tended to stick out from the skin [198].

The second study conducted insertion tests of both coated MNs and IRMNs on ex vivo dog ear skin without hair removal for 10 s using thumb pressure and then attached to the ear for 15 min [199]. The puncture performance and insertion efficacy for both coated and IRMNs were 100%; however, the coated MN patch was unsuitable, as the adhesive patch surrounding the coated MNs could not adhere sufficiently to the skin. This caused the needles to be lifted out of the skin after initial insertion, which corresponded to the poor delivery efficiency of coated MN (0.6%). This was not the case with the IRMNs, which showed 95% delivery efficiency within 10 s of needle application, even without prior hair removal from the skin. Whilst the delivery efficiency may not be as important for a vaccine, where only enough vaccine must be delivered to trigger the immune response, drugs with a narrow therapeutic window will require a specific dose to be delivered transdermally. The IRMNs in this study delivered almost all of the coated vaccine cargo without a large amount of waste, unlike the coated MNs. Furthermore, leaving some drug on the skin surface, as was the case with coated MNs in this study due to improper insertion, may be considered unsafe for specific compounds. Although IRMN technology has clear potential by taking advantage of the mechanical properties of the MNs, the technology must be refined to ensure complete needle deposition occurs.

Another strategy to ensure complete MN insertion without retraction out of the skin is to use “implantable MNs.” These MNs have been investigated particularly for scenarios where sustained release would be advantageous to the system. For example, one study detailed the effectiveness of an influenza vaccination from “patch-free” chitosan MNs, which consisted of chitosan MNs loaded with vaccine, with a dissolving supporting array (PVA/PVP), which gave extra length to the MNs to allow for complete insertion. The dissolvable supporting array was dissolved within the skin during insertion [200]. MNs were applied to the dorsal skin of ICR mice and Sprague–Dawley rats, and to the ear skin of crossbred LYD pigs to ensure complete insertion over a range of skin thicknesses. Following a 15-min insertion time, the supporting array fully dissolved, leaving the MNs fully embedded in the skin (at a depth of 800 µm in rat skin). All microscopic conduits created from MN insertion gradually repaired and disappeared within 8 h; thus, skin healing was not delayed. The MN-induced immune-enhancing effect from this MN system lasted for at least 16 weeks.

**Fig. 16:**
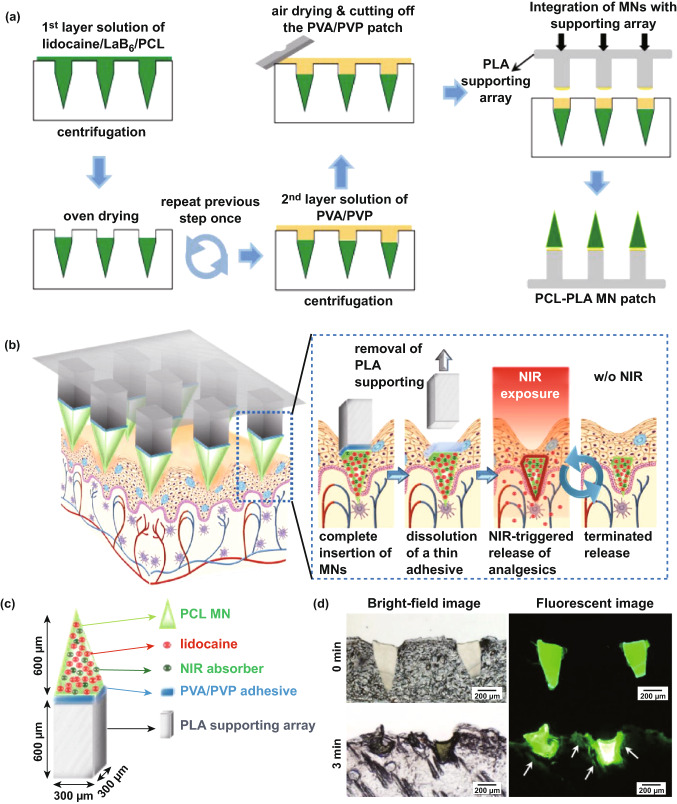
**a** Schematic illustrations of the fabrication process for the implantable polycaprolactone/polylactic acid (PCL/PLA) MN patch. **b** Schematic illustrations of the implantable microneedles (MNs) with near-infrared light triggerable properties. **c** Schematic illustrations of the implantable MN system and its composition, containing both the drug and near-infrared light absorber. **d** Histological sections of rat skin after insertion of coumarin 6-loaded MNs and then exposure to a near-infrared laser for 0 and 3 min. The arrows show the diffusion of the released coumarin 6 into the skin. PVP: polyvinylpyrrolidone; PVA: polyvinyl alcohol. Reprinted from Ref. [193] with permission from Royal Society of Chemistry

In another study, implantable MNs were formulated using a photo-triggerable system for patient-controlled analgesia (Fig. 16), whereby near-infrared light could be used to release the analgesic [193]. Near-infrared absorbers and analgesics were combined with a PCL/PLA supporting base array. The supporting base array was fabricated by casting molten PLA pellets onto a previously constructed polydimethylsiloxane mold. The polydimethylsiloxane mold was fabricated by a precision electrical discharge machining technology using a stainless-steel master structure consisting of 81 (9 × 9) tiny structures with center-to-center spacing between adjacent 1000-µm needles. The “removable design” of the supporting base array enabled the rapid implantation of the MNs into the skin to act as a drug depot, therefore reducing patch application time. Following irradiation with near-infrared light, the near-infrared absorbers in the implanted MNs absorb light energy and induce a phase transition to release the analgesic. Not only does this system allow for “on-demand” analgesia, the duration and modulation of lidocaine release were found to be controllable by varying the irradiation time and switching between the “on” and “off” status of the laser. Lidocaine was found to be released into the bloodstream within 10 min of MN application, and MNs achieved 95% bioavailability compared to a subcutaneous injection of the same drug. Whilst this may remove the requirement for multiple injections to control pain and may enable patients to control their pain more conveniently and comfortably, near-infrared light instruments are not commonplace within healthcare, and a patient would need both the MN patch and such an instrument to facilitate drug release. This takes away somewhat from the purpose of MNs—to be small, simple and convenient to use. Therefore, it is unlikely that such devices will become commonplace within health care until near-infrared light devices become commonplace, though the technology does illustrate that sophisticated MN devices may be formulated to tailor drug release to the individual patient.

Implantable MNs have also been used for sustained glucose sensing and insulin delivery [201, 202]. Powder-carrying MNs were developed which lacked a reconstitution step, allowing one to implant insulin powder directly without the requirement of an adhesive patch [202]. This allows delivery of the required dose of insulin without degradation issues, a common problem associated with its delivery using dissolving MNs. Insulin powder was encased in a CMC microshelled structure. Low fracture forces were observed at 5% CMC, indicating the breakage of the outer microshell, which occurred at the base of the structure. This was thought to potentially cause insertion difficulties via collapsing of the entire structure; thus, 10% CMC was used for studies thereafter. However, increasing CMC concentration increased the thickness of the microshells due to reduced shrinkage of the shells during the drying process, reducing the loading capacity of drug [202].

**Fig. 17:**
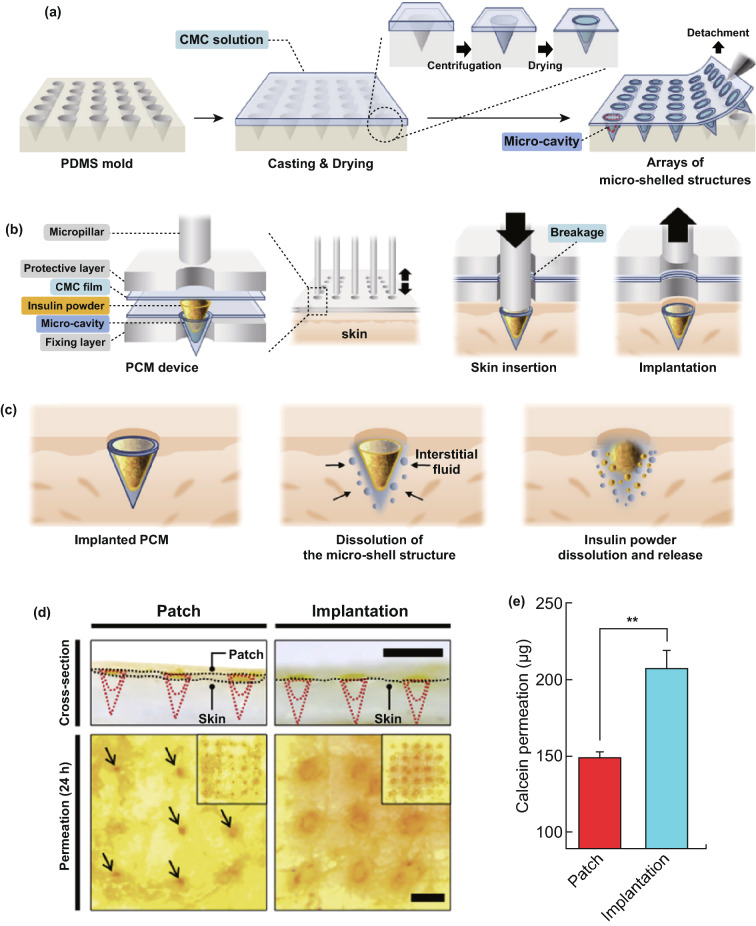
**a** Schematic illustration of fabrication of the insulin-loaded powder-implantable MNs. **b** Schematic illustration of the process of skin implantation using the micropillar-based implantation system. **c** Once inserted into the skin, dissolution of the outer part of insulin-loaded powder MNs occurred due to contact with interstitial fluid, allowing for insulin powder dissolution and release **d** Cross sections and skin surface images after 24 h of permeation using calcein-loaded powder-carrying MNs (dotted red lines) using a patch or an implantation system. The patch group showed incomplete implantation of the MNs, whereas the implantation group showed perfect implantation of MNs; therefore, no calcein powder was left on the skin as was the case with the patch group (black arrows). **e** Comparisons of the amount of permeated calcein between the patch and the implantation group after 24 h. Reprinted from Ref. [202] with permission from Elsevier

Powder-carrying MNs applied using a patch were not fully embedded into the skin, and a gap of 85 µm was observed between the tissue and the MN arrays. Complete skin insertion of the powder-carrying MNs only occurred when needles were applied using a micropillar-based implantation system (Fig. 17) [202]. Calcein was used as a control drug, and permeation of calcein was 147 and 206 µg for the patch group and implantation group, respectively [202]. Whilst the results are positive and indicate that powder insulin delivery was possible from this system, similarly to the near-infrared system, patients with diabetes will not have access to a micropillar-based implantation system, and thus, the results from this study are not necessarily translatable to real-life use [202]. Currently, patients commonly use patches for transdermal drug delivery applications. Thus, the implantation device used in this study would need to be accessible for all patients for this implantable, powder-carrying MN system to be viable for commercial use.

Bioinspired design, e.g., those that mimic animal organs, can also be used to improve the performance of MNs to successfully insert into skin. To prepare such complex structures, while still maintaining a high resolution, a technique often employed is direct laser writing (DLW) [203, 204]. This 3D microprinting process uses focused laser pulses to trigger highly localized polymerization reactions in a photoresist due to a two-photon absorption phenomena. By moving the laser focus in space, one can rapidly and reliably obtain elaborate fully 3D micro architectures with resolutions in the order 100 nm, which cannot be obtained with other microfabrication techniques, in a single fabrication step [205]. In light of this, by using DLW, an array of 80-µm-tall pyramid-shaped MNs was prepared with a micropatterned surface inspired by insects of the *Heteroptera* group (European true bug) [206]. Such a surface, which comprises 45° tilted microcones, is able to carry fluids unidirectionally along the cones’ direction, even against capillary forces. According to the authors, such structures could facilitate the injection of liquid media under the *stratum corneum* by directional external flow; however, no experimental evidence was provided in this study.

**Fig. 18:**
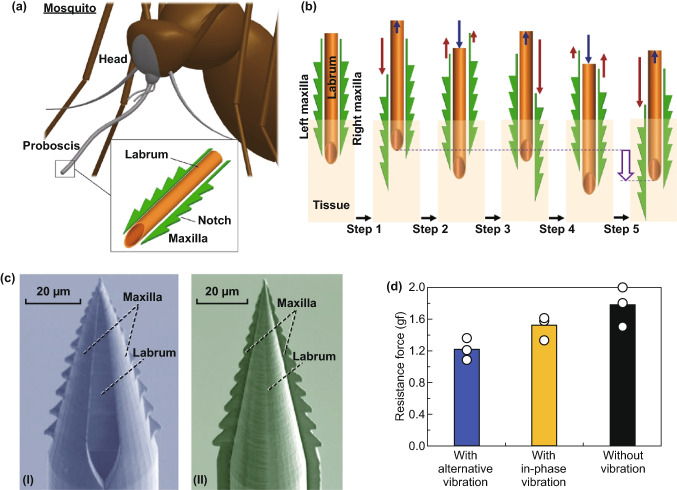
**a** Mosquito proboscis has a hollow labrum and two harpoon-shaped notched maxillae. **b** The incremental motion during the proboscis insertion reduces insertion force and surrounding tissue deformation. Reprinted from Ref. [208] with permission from Nature. **c** Magnified SEM images of microneedles made of IP-Dip polymer: (I) front view and (II) back view. **d** Resistance force under each vibration condition when the displacement is 0.8 mm. Circle indicates each measured value, and height of bar indicates the averaged value. Reprinted from Ref. [207] with permission from Inderscience

In another study, by using 3D printing technology, Suzuki et al*.* tried to mimic the structure of a proboscis of a mosquito to achieve painless insertion of hollow needles in the skin [207]. The proboscis is composed of different parts (e.g., labrum and maxillae), which mosquitos are able to move separately to penetrate the *stratum corneum* (Fig. 18). In the study, the authors fabricated a 100-µm-wide bioinspired needle composed by two separate parts, which could vibrate independently and showed that such design could reduce the force necessary to insert (and remove) the needle. Moreover, the capillary force generated inside the device was sufficient to draw enough blood to perform glucose analysis in few seconds. While this is not the first time that the proboscis architecture has been replicated by microfabrication techniques, the use of DLW allowed for an unprecedented 3D freedom during the fabrication process [207].

While bioinspired structures possess metaproperties, which can improve the performances of MNs, DLW can be used to realize standard needle designs without the complexity typically associated with microfabrication, such as cleanroom facilities, expensive equipment, and multi-step procedures [209, 210]. For instance, an injection device comprising an array of hollow MNs connected to a 2-mm^3^ reservoir was fabricated, all was printed in a single step using a biocompatible resin [16]. Due to the flexibility of DLW, the authors could optimize the dimensions of the needles to improve skin penetration and flow rate of the drug and successfully tested their working devices on mice [16].

Although DLW brings the advantages of 3D printing to the microscale, only polymeric materials can be printed with adequate resolutions. This often limits the nanowires efficacy because of their mechanical properties (Young modulus < 3.5 GPa) [211]. However, DLW can also be employed to realize template prototypes for mold-casting fabrication, overcoming the material constraints. This rapid prototyping technology can be useful for optimization studies which require testing of different designs and/or materials in a short amount of time [212]. Recently, such an approach was used in a comparative study [127]. The performances of various MNs heights, shapes, and separation on the penetration and drug-releasing characteristics of different biodegradable polymer blends were examined. It was shown that 900 µm conical and square-pyramidal shapes showed the best insertion performances (up to 90% of their total length) and, consequently, could deliver the highest amount of drug by MN dissolution [127].

## Conclusion and Outlook

Controlled drug delivery has become a promising area of research and development over the last decade. Several clinical studies exhibited that MNs cause less pain compared to hypodermic needles [30, 31, 32, 33, 138]. However, the crucial characteristic of MN technology is the capability of piercing the *stratum corneum* without breaking or bending during insertion. This ensures that the release of active compounds from MN patches occurs at the predetermined place and time.

Furthermore, mechanical tests using different insertion assays and a wide variety of skin models are carried out to predict the likelihood of successful needle insertion. Such analysis is performed prior to clinical practice to allow researchers to refine and improve upon the insertion process.

Implantable and insertion-responsive MNs (e.g., with micropillar or pedestal) are a sophisticated use of MN technology, compared to without pedestal-based platforms However, solid MNs have been largely replaced due to their “two-step” design by the use of “one-step” MNs (i.e., dissolving, hydrogel-forming). The use of implantable and insertion-responsive MNs appears in some cases to require a separate piece of equipment for successful insertion, such as a micropillar, or a near-infrared light device. Additionally, current fabrication methods used to prepare pedestal-based patches are time-consuming. To circumvent this hurdle, 3D printing methodologies such as direct laser writing (DLW) can pave the way for quicker and more precise fabrication of separatable MN patches [127, 213]. Compared to other microfabrication technologies, DLW offers several advantages: It can achieve 3D structures with arbitrary complexity and sub-micrometric resolutions; it allows an extremely flexible printing process, where design and printing parameters can be changed (and tested) rapidly; it does not necessarily require cleanroom facilities; and its fabrication methodologies are intrinsically fast, thus making it a promising technology when considering the eventual need to scale up MN production on an industrial level [214]. However, this technique can only reliably print polymeric materials (usually acrylates and epoxide-based resins), which may not reach high Young moduli (˃ 5 GPa) and are not always biocompatible. While these limitations may be considered difficult to overcome, one must also consider that polymeric materials and composites have properties and functionalities that can be easily tuned over a broad range of possibilities. These materials can be optimized to fit one’s needs by means of chemical synthesis and design [203]. Moreover, the use of 3D-printed microstructures to serve as a template for mold-casting methods can surpass the materials limitations altogether, combining the advantage of DLW resolutions with the properties of otherwise unprintable materials [5].

This review clearly details the range of literature that explores factors which influence the ability of MNs to effectively insert into the skin. Utilizing pillars as a separate applicator can be one solution to fully insert the MNs into the skin by consistent spreading of the force onto each MNs, though this somewhat removes from the simplicity and convenience associated with MNs. Several other aspects including needle geometry and density/mechanical properties, distance between two needles, and the employed materials must be considered to fabricate MNs for successful insertion. Such factors may be tailored to achieve the appropriate level of MN penetration required for successful drug delivery or specific site targeting. Therefore, altering the MN design, geometry and fabrication material can affect the needles ability to overcome skins natural elasticity and pierce the skin, all of which can be tailored to maximize drug delivery on a case-by-case basis. More recently, and in the future, computational models may become more popular to predict the optimal MN design for transdermal delivery of a specific drug [18, 145]. Assuming that computational models may be considered accurate, this would remove the need to source ex vivo skin tissues, an advantage when considering that MNs must be accepted by regulatory authorities to become a clinical success, and eliminating the need for ex vivo skin tissue for mechanical testing makes regulatory acceptance more likely since such samples cannot be used for quality control (QC) purposes.

In regard to regulatory authorities, one must understand the basic requirements of MNs in order for them to make the transition from benchtop to bedside. At their most basic level, MNs must be able to reliably insert into the skin for transdermal drug delivery. Thus, regulatory authorities must decide on what constitutes “sufficient MN insertion” so the appropriate QC tests can be designed for testing prior to MN release to the public. Furthermore, as no obvious sensation occurs from the application of a MN array, some indication of correct application and delivery may be required for the patient [215, 216]. A pressure-indicating film has been designed for such a purpose, whereby a color-changing film was successfully incorporated into a MN array to indicate successful MN insertion, providing visual feedback to the end user [217].

Ultimately, regulatory authorities will need to decide on the acceptance criteria for MNs—of which, successful insertion is just one aspect. MNs hold great potential for the transdermal delivery market, and once such regulatory hurdles are overcome, the benefits for both patients and the pharmaceutical industry will be substantial.
